# LignAmb25: A comprehensive AMBER force field addressing lignin’s structural and chemical diversity

**DOI:** 10.1016/j.bpj.2026.04.004

**Published:** 2026-04-07

**Authors:** Marco Lapsien, Michele Bonus, Julian Greb, Holger Gohlke

**Affiliations:** 1 Institute for Pharmaceutical and Medicinal Chemistry, Heinrich Heine University Düsseldorf, 40225 Düsseldorf, Germany; 2 Bioeconomy Science Center (BioSC), Heinrich Heine University Düsseldorf, 40225 Düsseldorf, Germany; 3 Institute of Bio- and Geosciences (IBG-4: Bioinformatics), Forschungszentrum Jülich, 52425 Jülich, Germany

## Abstract

LignAmb25 is a comprehensive force field for lignin molecular-dynamics simulations implemented natively within the AMBER package. The force field includes parameters for all common monolignol units (*p*-coumaryl, coniferyl, caffeyl, and sinapyl alcohol) and their associated linkages (β-O4, β-5, β-β, β-1, 5-5, 5-O4, α-O4, benzodioxane [BDO], and dibenzodioxocin [DBDO]) along with less commonly encountered units such as tricin, spirodienones, and hydroxystilbenes. This enables simulations of both softwood and hardwood lignin structures with compositions that would be difficult to isolate experimentally. Force-field parameters were initially derived from the GAFF2 force field and systematically optimized using quantum-mechanical calculations at the ωB97X-D4/def2-TZVPP level of theory on conformer ensembles derived via the Conformer-Rotamer Ensemble Sampling Tool/Commandline Energetic Sorting (CREST/CENSO) conformational sampling toolchain. Partial atomic charges were derived using the restrained electrostatic potential (RESP) methodology, consistent with AMBER conventions. Experimentally measured crystal structures of lignin simulated with LignAmb25 accurately retain their packing based on calculations of the root-mean-square deviation (RMSD) and density error compared to the deposited crystal structure, thereby exceeding the performance of the lignin force field for CHARMM. Additionally, LignAmb25 is shown to reliably estimate the enthalpy of vaporization and the absolute hydration free energy of lignin-related compounds. The LignAmb25 force field is provided in two variants: LignAmb25^Solo^, a standalone version not meant for use with other biomolecular force fields that focuses on accurate modeling of lignin-solvent interactions, and LignAmb25^HF^, a version that is compatible with all other major biomolecular force fields in the AMBER molecular-dynamics suite. This includes force fields of the GLYCAM (carbohydrates), ff19SB (proteins), and LIPID (lipids) families, as well as the DNA and RNA force fields routinely used in AMBER. The LignAmb25 force field will be distributed as of AMBER 26.

## Significance

Lignin, a complex aromatic heteropolymer, remains one of the most challenging biopolymers to characterize experimentally due to its structural heterogeneity and lack of a defined primary sequence. Wet-lab analytical methods face significant limitations due to lignin’s poor solubility and tendency to aggregate. These experimental challenges make computational approaches essential for understanding lignin’s physicochemical properties and advancing lignin valorization. We present LignAmb25, a molecular mechanics force field for lignin implemented within AMBER, enabling researchers to investigate lignin structures and dynamics under conditions difficult to access experimentally. LignAmb25 integrates into the AMBER force-field system and improves upon existing lignin force fields by including spirodienones and hydroxystilbenes as less commonly encountered monolignol units and the addition of several new linkages.

## Introduction

Valorization of biomass has gained increasing attention due to its abundance and the negative impact on climate of established procedures for obtaining raw materials (1,2). Biomass is primarily composed of plant cell wall tissue, which itself is composed of the polysaccharides cellulose and hemicellulose, as well as lignin (3). The latter is a complex three-dimensional heteropolymer that provides structural integrity to the plant cell wall (4). Depending on the biomass source, lignin makes up to between 15 and 40% of the plant cell wall mass (5,6), a noticeable fraction that is most often simply burned and, therefore, not properly valorized (7). Combating this waste of lignin is crucial, and depolymerization of lignin can yield sought-after functionalized aromatic compounds, such as vanillin, which is heavily used in the food industry (8), syringaldehyde, which is associated with the treatment of bacterial infections (9), or phenol, which can be used to build bio-based resins (10). Despite progress in valorization strategies, lignin’s full potential remains largely untapped. Deciphering its recalcitrance against depolymerization and selective chemical conversion is key to replacing petroleum-derived aromatics, such as benzene, toluene, and xylenes with renewable alternatives (11). Additionally, lignin plays a crucial role in cellulose-based textile production, where its presence negatively impacts yields (12) and spinning efficiency (13), both of which could likely be improved by a better understanding of lignin’s conformational dynamics and solvent interactions. Despite its potential, the molecular-level understanding of lignin’s behavior remains limited, hindering rational design of extraction processes, enzymatic degradation strategies, and lignin-based materials (14). The structural complexity of lignin presents unique analytical challenges that distinguish it from other biopolymers (15). Unlike proteins and nucleic acids, lignin lacks a defined primary sequence and exhibits substantial heterogeneity in both monomer composition and interunit linkage patterns due to the nature of its biosynthesis (16,17). Lignin biosynthesis occurs via the coupling of oxidized monomer radicals and is therefore driven by stochastic variance (18).

Molecular-dynamics (MD) simulations present a powerful approach to overcome the experimental limitations associated with lignin research and enable the investigation of lignin at atomic resolution. Computational studies have provided insights into lignin’s interactions with cellulose (19), conformational preferences of individual linkages (20), and passive transport across membranes (21). However, the accuracy of these simulations depends critically on the quality of the underlying force-field parameters. While a lignin force field has been developed for Chemistry at HARvard Marcomolecular Mechanics (CHARMM) (LF-CHARMM) (22,23), the Assisted Model Building with Energy Refinement (AMBER) (24) MD package, widely used for biomolecular simulations, has lacked dedicated lignin parameters optimized using AMBER-consistent methodologies. The two frameworks differ fundamentally in their approach to charge derivation: CHARMM/CGenFF refines HF/6-31G^∗^ electrostatic potential (ESP) charges against explicit water interaction energies, directly optimizing solvation behavior (25), whereas AMBER/restrained electrostatic potential (RESP) relies on the systematic overestimation of dipole moments at the HF/6-31G^∗^ level to implicitly account for condensed-phase polarization (26). For lignin, which combines aromatic rings capable of π-π stacking with polar hydroxyl and methoxy substituents, these differing philosophies may lead to divergent predictions of hydrogen-bonding patterns, aggregation behavior, and conformational preferences in aqueous and non-aqueous environments.

The development of accurate force-field parameters for lignin presents unique challenges. The diversity of linkage types requires careful parameterization of numerous chemical environments that govern the three-dimensional structure (15,27). To address this, we present LignAmb25, a comprehensive force field for lignin developed specifically for the AMBER MD suite. Building upon the General Amber Force Field (GAFF2) (28) framework, we systematically optimized parameters using extensive quantum-mechanical (QM) calculations and conformational sampling via the CREST/CENSO (29,30) conformational search pipeline. Our parameterization strategy focused on providing a modular force field that provides the (to date) most extensive set of linkages and monomers described in the literature. Furthermore, we prioritized integration with other existing biomolecular force fields implemented in AMBER, such as ff19SB (31), GLYCAM06 (32), LIPID21 (33), and the nucleic acid force fields (34,35), routinely used to perform MD simulations.

The integration of LignAmb25 with existing AMBER force fields is particularly important for studying lignin-carbohydrate complexes (36), lignin-enzyme interactions (37), and the behavior of lignin in various solvent systems relevant to biorefinery processes (38). By providing these parameters to the community through the standard AMBER distribution, we aim to facilitate computational studies that can guide experimental efforts in lignin valorization and utilization.

## Materials and methods

### Determination of force-field scope

**Figure 1:**
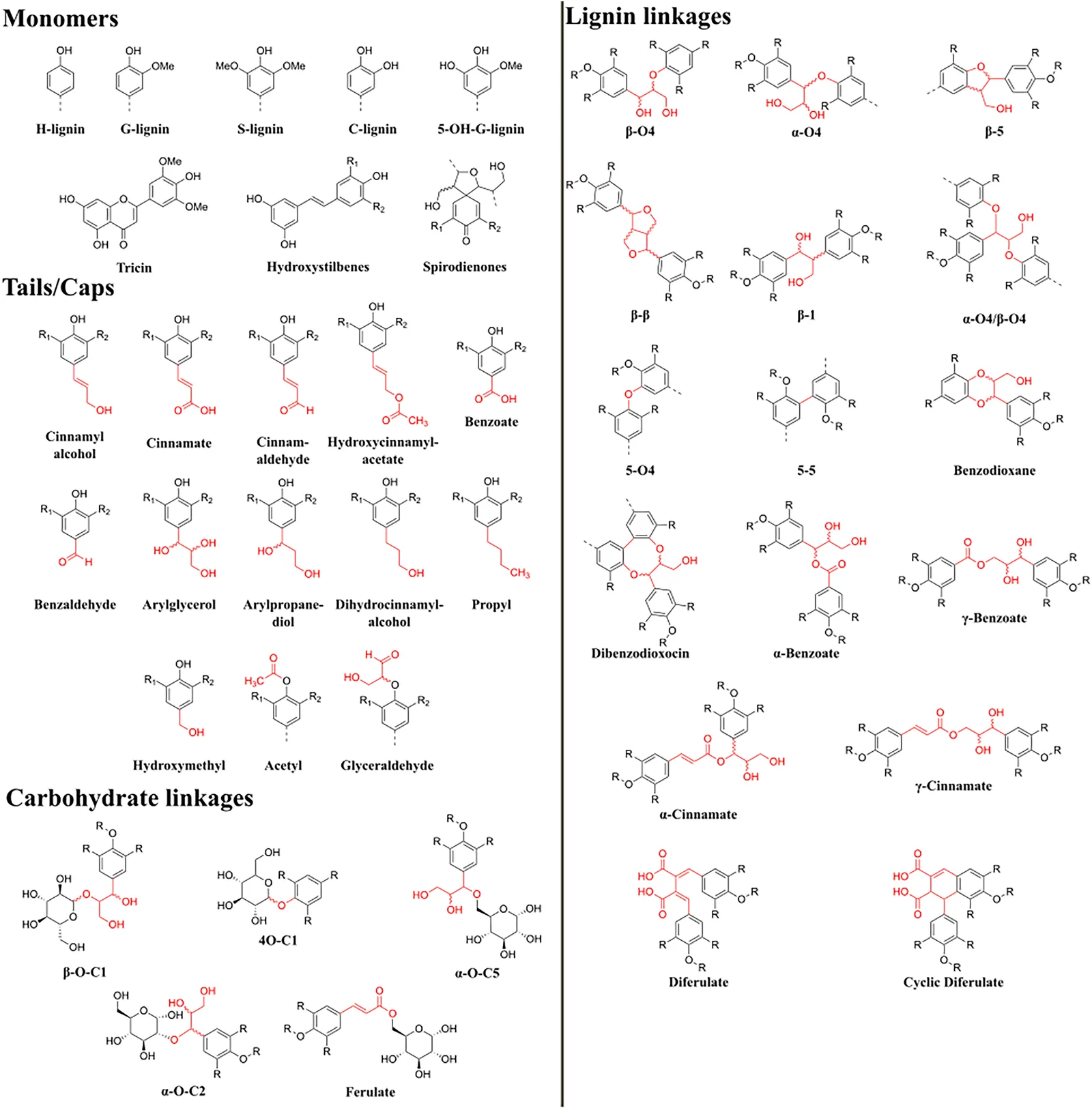
Overview of monomers, end groups, and linkages included in LignAmb25 Apart from abundant monolignols, the less common 5-OH-G-unit (39), tricin, hydroxystilbenes, and spirodienones were included. Each linkage, except for the spirodienone constructs, has dedicated fragments per stereoisomer (*wavy bonds*). Linkages between lignin and carbohydrates can exclusively be modeled with force fields from the GLYCAM family.

The choice of chemical species to include in the force field was based on experimentally observed monolignols and linkage types (Fig. 1). This comprises the predominant coumaryl- (H), guaiacyl- (G), and syringyl- (S) lignin (15,27) but also the less commonly found caffeyl- (C) lignin (39), and non-traditional monolignols such as the flavonoid tricin (40), as well as spirodienone (41) and hydroxystilbene (42) constructs. The varying chemical properties of the monolignols, coupled with the radical nature of the lignification process, lead to a variety of linkages found in lignin polymers. The β-O4, β-5, β-β, 5-O4, 5-5, and α-O4 linkages are among the more commonly encountered linkages (15,27). However, less frequently encountered linkages, such as the benzodioxane (BDO) (43) and dibenzodioxocin (DBDO) (44) as well as α-cinnamate/-benzoate (45) and γ-cinnamate/benzoate (46,47) linkages, were also included in the parametrization process. Additionally, linkages between lignin and carbohydrates were considered by including the β-O-C1, 4O-C1, α-O-C5, α-O-C2, and ferulate linkage types between monolignols and monosaccharides (45,48).

### Definition of force-field fragments

**Figure 2:**
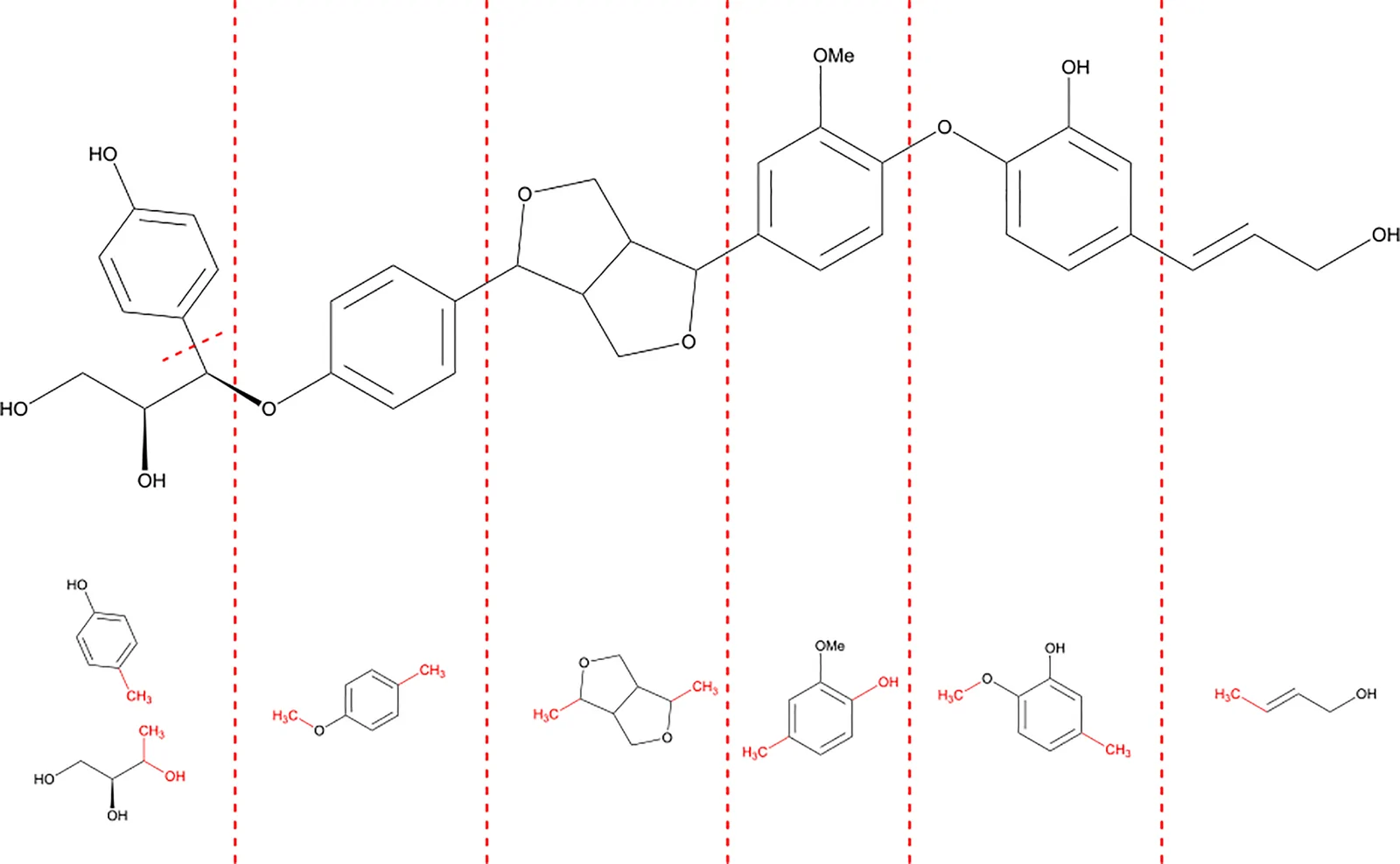
Capping strategy chosen for LignAmb25 development The fragmentation of an example polymer into force-field fragments is shown. Fragmentation points were chosen to maximize modularity of the force field while respecting established guidelines for RESP charge cap groups where possible (49). Most interunit linkages consist of ether bonds and carbon-carbon single bonds. Ether bonds were fragmented using a methyl group on the fragment carrying the oxygen and a hydroxyl group on the other fragment and applying an intermolecular charge constraint of 0 between the two groups. Carbon-carbon single bonds were fragmented by attaching a methyl group to each fragment and applying an intramolecular charge constraint of 0 per capping group. As modularity of the force-field definition was given a high priority, conjugated systems sometimes had to be fragmented too (*bottom right*), which might impact the accuracy of charge derivation.

Next, lignin dimers, trimers, and tetramers covering the pool of linkages included in the parametrization process were built, and a set of force-field fragments was obtained by splitting the multimers at bonds connecting to the aromatic ring of a monomer (Fig. 2). This approach was chosen as it reduces the number of fragments needed to model individual linkages, hence, keeping the total number of fragments reasonably low relative to lignin’s chemical complexity. This approach yielded distinct fragments for each linkage (with the exception of the 5-5 and 5-O4 linkage as a single bond separates the two aromatic rings) and separate monolignol fragments. In the case of H-, G-, S-, and C-lignin, they consisted only of the aromatic ring with a varying number and position of open valences depending on whether the ring is a branching point or part of a linear chain. Additionally, a variety of “tail” fragments was obtained that encapsulate the variations of monolignols at the C1 position. Although stereochemistry is often neglected in lignin research, a dedicated fragment of each stereoisomer per linkage was created, and, in the case of the β-O-C1 and 4O-C1 lignin-monosaccharide linkages, variants specific to the α- and β-anomers were built.

### Residue and atom type naming conventions

Residue names were chosen to avoid conflicts with existing biomolecular force fields in AMBER. This, unfortunately, limited the available three-character codes, resulting in non-trivial character combinations for most fragments. Aromatic fragments usually have the number of open valences as the first character, the type of monolignol as the second character, and a dedicated character that describes the position of the open valences, e.g., the code “1H1” corresponds to an H-unit with one open valence at the C1-position. Non-aromatic fragments mostly do not follow such a scheme and were instead assigned residue names not occupied by other force fields. Fragments with multiple stereoisomers share the same first two characters and differ in the third character to distinguish them. The letters E and T (in uppercase and lowercase, denoting one enantiomer each) represent the relative configurations *erythro*- and *threo*, where possible. Compounds with a single stereocenter will have R and S as the third letter in agreement with Cahn-Ingold-Prelog (CIP) nomenclature.

As atom types in the AMBER format only allow for two characters, the number of non-overlapping terms was even more sparse than for residue names (Table S1). The first character of an atom type corresponds to the element it describes (e.g., O for oxygen). Carbon atom types are an exception to this, as they are divided into aromatic (lowercase c) and non-aromatic (uppercase C) carbon atoms. The second character of each atom type was chosen to avoid overlaps with existing biomolecular force fields in AMBER.

### Conformational search

Representative ensembles of conformers for force-field fragments and lignin multimers were derived using the Conformer-Rotamer Ensemble Sampling Tool/Commandline Energetic Sorting (CREST/CENSO) conformational search pipeline. Initial models were generated using the RDKit Python package (version 2025.03.1) (50) and pre-optimized using the Geometry, Frequency, Noncovalent, second generation (GFN2) (51) method integrated into the extended Tight Binding (xTB) method (version 6.6.1) (52) in implicit water using the Generalized Born Surface Area (GBSA) solvation model (53). The resulting geometries were used as starting points for a conformational search using CREST (version 3.0.2) applying the Geometry, Frequency, Noncovalent Force Field (GFNFF) (54) method to avoid topology changes and the GBSA water model. The CREST conformer ensembles were subsequently filtered using CENSO (version 2.1.3) to obtain a representative Boltzmann-weighted set of conformers. Details on the settings for each CENSO stage can be found in the supporting material (Table S2).

### RESP charge derivation

To obtain optimized partial atomic charges, a RESP fit (26) was performed. Capping groups per fragment were chosen based on best practices recommended by the developers of the RESP ESP charge Derive (RED) server (49,55). As a consequence of the approach chosen for defining force-field fragments, they are primarily used to accommodate the splitting of ether and carbon-carbon single bonds (Fig. 2). The former were split by attaching a hydroxyl capping group to one fragment and a methyl capping group to the other. To ensure compatibility with the GLYCAM force-field family (32), the hydroxyl capping group was constrained to a charge of −0.1940, whereas the methyl capping group was constrained to the opposite charge of +0.1940. This ensures charge neutrality when lignin-carbohydrate linkages are built. The carbon-carbon single bonds were split by attaching a methyl capping group to each fragment and applying an intramolecular charge constraint of 0 to either capping group.

The molecular electrostatic potential (MEP) per conformer of the CENSO-derived ensemble per force-field fragment was calculated at the HF/6-31G^∗^ (56,57,58), MP2/cc-pVTZ (59,60), B3LYP/6-31G^∗^ (61), B3LYP/cc-pVTZ, and ωB97X-D/cc-pVTZ (62) level of theory using Gaussian16 (63) with four shells built around the molecule in increments of 1.4, 1.6, 1.8, and 2.0 times the van der Waals radius according to the standard RESP procedure (26). The grid point density was increased to 17 points per Å^2^, and six different orientations per conformer were used to reduce the orientation dependence of the resulting RESP charge set (Fig. S1) (64). The MEP of lignin multimers was calculated in the same manner with only one orientation used per multimer.

The RESP fit was carried out using PyRESP (65) with the input files prepared using PyPE_RESP (66). Charge fitting was performed according to the standard two-stage procedure, where, in the second stage, only methyl groups were reoptimized. The additive RESP model with varying hyperbolic charge restraint weights (0.0003, 0.0005, 0.0007, and 0.001) in the first stage and a fixed hyperbolic charge restraint of 0.001 in the second stage was applied. Conformers were fit together, with each conformer weighted according to the Boltzmann weight determined during the CENSO runs. Since six different orientations per conformer were used, the weight per orientation was equal to the conformer Boltzmann weight divided by 6. Mean Relative Root Mean Square Error (RRMSE) values obtained for RESP fits at different levels of theory and charge restraint weights are given in Table S3.

### Bond-stretching parameter optimization

The lowest-energy conformer of each lignin multimer was used to perform potential energy surface (PES) scans using the r2SCAN-3c (67) composite method implemented in ORCA (version 6.0.0) (68). All bonds in each structure were scanned in steps of 0.02 Å in a range of ±0.4 Å of the equilibrium bond length measured in the conformer geometry (41 steps total). Single-point energy calculations at the ωB97X-D4/def2-TZVPP (69,70,71) level of theory were performed on each scan geometry to obtain the final energy profile. Detailed information on convergence settings is provided in the supporting material (Table S4). PES energy profiles (*E*_*bond*_) were fitted to the AMBER bond-stretching potential energy term using the 17 points closest to the equilibrium (±8 steps) (Eq. 1):

(1) $$\begin{array}{c} E_{bond}=k{\left(r-r_{eq}\right)}^{2} \end{array}$$

where *k* is the force constant, *r* is the scanned bond length, and *r*_*eq*_ is the equilibrium bond length. Bonds between identical atoms in different molecular contexts were grouped together based on atom types. Where necessary, these groups were further divided into subclusters using Density-Based Spatial Clustering of Applications with Noise (DBSCAN) clustering based on force constant and equilibrium length variance, and a mean force constant and equilibrium length were derived for each (sub)cluster.

To assess improvement over GAFF2 parameters, single-point energy calculations on each PES scan geometry were performed using sander with the target bond term parameters nulled, changed to the mean cluster parameters, and changed to the GAFF2 parameters, respectively. The bond term contribution for the fitted and GAFF2 parameters was calculated by subtracting the single-point energies obtained with the bond stretching term nulled from the single point energy obtained with the respective bond parameters enabled. The performance of the two parameter sets was assessed using the mean absolute error against the PES energy curve, with a decrease of at least 5% in the mean absolute error considered better and an increase of at least 5% considered worse. This threshold was chosen to be conservative relative to the intrinsic numerical variability of the QM reference data, which at the ωB97X-D4/def2-TZVPP level of theory with tight SCF convergence criteria is expected to be negligible while remaining sufficiently large to distinguish meaningful improvements from noise inherent to the parameter fitting procedure.

### Angle-bending parameter optimization

Parameters for angle bending were derived similarly to those for bond stretching, with PES scans performed in 2° increments over a range of ±40° from the equilibrium bond angle in the lowest-energy conformer geometry (41 steps total). Detailed information on convergence settings is provided in the supporting material (Table S4). The form of the objective function was according to the AMBER angle-bending potential energy term (Eq. 2):

(2) $$\begin{array}{c} E_{angle}=k{\left(\theta -\theta_{eq}\right)}^{2} \end{array}$$

where *E*_*angle*_ is the potential energy, *θ* is the scanned bond angle, and *θ*_*eq*_ is the equilibrium angle. Improvement over GAFF2 parameters was assessed similarly to bond-stretching parameters, including the previously fitted bond parameters in the parameter set.

### Torsion-angle parameter optimization

Torsion-angle parameters were derived by performing PES scans for each rotatable bond in each conformer of each lignin multimer in steps of 10° through a full 360°, starting from the equilibrium torsion angle measured in the conformer geometry (37 steps total). Detailed information on convergence settings is provided in the supporting material (Table S5). Torsional barriers (*V*), periodicities (*n*), and phase angles (*γ*) were derived using the differential evolution algorithm implemented in the evosax (version 0.2.0) (72) and JAX (73) Python packages with the objective function according to the AMBER torsion-angle potential energy term (Eq. 3):

(3) $$\begin{array}{c} E_{torsion}=\sum_{i=1}^{N}\frac{V_{i}}{2}\left[1+\cos\left(n_{i}\phi -\gamma_{i}\right)\right] \end{array}$$

where *E*_*torsion*_ is the torsional energy, *N* is the maximum number of terms in the cosine series, and *ϕ* the scanned torsion angle. A maximum of *N* = 4 terms per dihedral pattern targeting the rotated bond was allowed. Additionally, dihedral patterns were weighted according to the number of potentials that arise per rotatable bond, and the angle values of all torsion patterns upon rotation of the bond were considered in the fit. Phase angles were constrained to multiples of 30° (i.e., 0°, 30°, 60°, 90°, 180°, or 270°). Optimization employed a hierarchical search strategy with an initial screening (15 generations, population size 25) followed by a full optimization (80 generations, population size 40) of the top 10% of parameter combinations. Early stopping was applied when no improvement was observed for 20 generations.

A mean parameter set per molecule was obtained by selecting the fitted parameter set yielding the lowest Boltzmann-weighted Root Mean Square Error (RMSE) against the PES energy profiles of all conformers of the respective lignin multimer. The conformer-averaged parameter sets were then grouped by the atom types within the rotatable bonds. Subclusters were identified using hierarchical clustering with Ward linkage on pairwise RMSE distances between Boltzmann-weighted mean energy profiles with an RMSE threshold of 3.0 kcal mol^−1^ and a minimum cluster size of five members; smaller clusters were merged with their nearest-neighbor cluster. The best parameter set within each subcluster was identified by selecting the fitted parameter set yielding the lowest Boltzmann-weighted RMSE against the PES energy profiles of all conformers of all molecules in the cluster.

Improvement over GAFF2 parameters was assessed similarly to bond-stretching and angle-bending parameters, including the previously fitted bond and angle parameters in the parameter set.

### Translation of CHARMM force fields into AMBER

To evaluate the transferability of the CHARMM lignin-carbohydrate force field to the AMBER simulation engine, partial atomic charges for each lignin-related compound were rederived following the parametrization outlined in the LF-CHARMM showcase (23). Geometry optimizations were performed at the MP2/6-31G^∗^ level of theory with tight convergence criteria using Gaussian 16 (63). Partial atomic charges were obtained by fitting to electrostatic potentials computed at the HF/6-31G^∗^ level of theory on a grid of water-probe positions shifted 0.2 Å from the molecular surface, with a QM-to-MM scaling factor of 1.16. Bonded and Lennard-Jones parameters were taken directly from LF-CHARMM. CHARMM topology and coordinate files were converted to the AMBER-compatible CHAMBER format using parmed (74). The CHAMBER format preserves the full CHARMM functional form within the AMBER prmtop files, including Urey-Bradley terms, improper dihedrals, and CMAP corrections where applicable. Simulations with CHARMM parameters used CHARMM 1–4 scaling conventions (Scaling factor for 1--4 Electrostatic interactions [SCEE] = 1.0, Scaling factor for 1-4 Non-Bonded interactions (SCNB) = 1.0), and employed a force-switching function for Lennard-Jones interactions transitioning between 10 and 12 Å.

### Unbiased MD simulations

All MD simulations described in this work were performed using the baseline version (gas-phase simulations), MPI-accelerated (minimization and equilibration), and GPU-accelerated version (production simulations) of the pmemd MD engine as part of AMBER24 (24,75). Simulations were done using either the newly developed LignAmb25 force field or the GAFF2. Partial charges associated with the GAFF2 force field were derived using the RESP protocol described above with MEPs calculated at the HF/6-31G^∗^ level of theory.

For additional details on relaxation, thermalization, and production simulations per system, see the respective section below. Trajectories were analyzed using cpptraj (76).

### Modeling and simulation of crystal systems

18 crystal structures of lignin-related compounds and lignin dimers were taken from the Cambridge Structural Database (77). The structures were previously simulated using LF-CHARMM and therefore allow direct comparison between LF-CHARMM and LignAmb25 (23). Unit cells of the crystals were taken as provided from the simulations using LF-CHARMM. The PropPDB and ChBox tools from AmberTools25 were used to extend unit cells to 50.0 Å in each dimension and to ensure that experimental unit cell dimensions are retained (24).

Three minimization steps with gradually decreasing harmonic restraints from 25.0 to 15.0 to 10.0 kcal mol^−1^ Å^−2^ on heavy atoms were performed using the steepest descent algorithm for 2,500 steps and the conjugate gradient algorithm for another 2,500 steps (5,000 steps total). The system was then thermalized over 200 ps from 80 K to the temperature at which the experimental data were collected under NVT conditions with harmonic restraints of 10 kcal mol^−1^ Å^−2^ applied to heavy atoms. Temperature scaling was controlled using Langevin dynamics with a collision frequency of 1.0 ps^−1^ (78). Six steps of length 100 ps each under NPT conditions were performed with gradually decreasing restraints on heavy atoms (10.0–0.01 kcal mol^−1^ Å^−2^) to adapt the system density to a pressure of 1 bar. Pressure was controlled using isotropic position scaling via the Berendsen barostat with a relaxation time of 1 ps (79). The total thermalization and density adaptation time was 800 ps. All bonds involving hydrogen atoms were constrained using the SHAKE algorithm (80), permitting an integration timestep of 2 fs. A non-bonded cutoff of 12.0 Å was applied for van der Waals and short-range electrostatic interactions. Long-range electrostatic interactions were treated using the particle mesh Ewald (PME) method (81). Production simulations were run for 20 ns over four independent replicas, with the 200 frames obtained from the last 10 ns of simulation time used for the calculation of mean crystal properties. The simulation time was chosen to be identical to the crystal simulations performed using LF-CHARMM to ensure comparability. Additionally, crystal simulations were repeated with a simulation protocol faithful to the LF-CHARMM work (23). In this regard, each system was first energy-minimized using 5,000 steps of steepest descent to resolve steric clashes from crystal packing. This was followed by a brief 2-ps NVT equilibration at the experimental crystallographic temperature using a Langevin thermostat with a collision frequency of 1.0 ps^−1^ and a 2-fs timestep with SHAKE constraints on hydrogen-containing bonds. Production simulations were then run for 20 ns in the NPT ensemble at 1 atm using isotropic pressure scaling, with the same thermostat settings. A 12.0-Å non-bonded cutoff was used with CHARMM-style van der Waals force switching between 10.0 and 12.0 Å. The last 10 ns of the 20-ns trajectory were considered for measuring changes in crystal structure.

### Calculation of the enthalpy of vaporization

To estimate enthalpies of vaporization, gas-phase simulations were conducted by placing a single copy of the respective molecule in the simulation box with no periodic boundary conditions applied and a long-range interaction cutoff of 99.0 Å set. The liquid state was mimicked by placing 500 copies of the respective molecule in a 60 × 60 × 60-Å cube using packmol (82) with periodic boundary conditions enabled. Long-range electrostatic interactions were treated using the PME method for all liquid-phase simulations with a real-space interaction cutoff of 10.0 Å applied (81). Energy minimization was performed in three stages of 10,000 cycles each (8,000 steepest descent followed by 2,000 conjugate gradient), with harmonic restraints on non-hydrogen atoms progressively reduced from 25.0 to 15.0 to 10.0 kcal mol^−1^ Å^−2^. Thermalization and pressure adaptation consisted of an initial 100-ps NVT simulation with heating from 100 to 298 K, followed by six 100-ps NPT stages (total time, 700 ps) to adapt the system’s density to a pressure of 1 bar. Positional restraints were gradually decreased from 10.0 to 0.01 kcal mol^−1^ Å^−2^ over the six NPT stages. Temperature was maintained at 298 K using a Langevin thermostat with a collision frequency of 2.0 ps^−1^, and pressure was controlled using the Berendsen barostat with isotropic position scaling and a relaxation time of 1.0 ps for liquid-phase simulations. In both the gas-phase and liquid-phase simulations, all bonds involving hydrogen atoms were constrained using the SHAKE (80) algorithm, permitting a 2-fs integration timestep. Production simulations were carried out for 10 ns over four independent replicas, with Δ*H*_vap_ calculated via Eq. 4:

(4) $$\begin{array}{c} \Delta H_{vap}=U_{g}-U_{l}+k_{B}T \end{array}$$

where *U*_*g*_ and *U*_*l*_ represent the average molecular potential energies in the gas and liquid phase, respectively, *k*_*B*_ denotes the Boltzmann constant, and *T* is the temperature in Kelvin. *U*_*g*_ and *U*_*l*_ were obtained by computing the average of the average molecular potential energy per replica. Statistical uncertainties were estimated using the Flyvbjerg and Petersen blocking transformation to account for autocorrelation in the energy time series (83).

### Calculation of hydration free energy

Absolute hydration free energies were calculated using a previously developed thermodynamic integration (TI) single-topology softcore annihilation approach (84). Solutes were solvated in TIP3P (85) water boxes (16-Å buffer), and decoupling was sampled in 11 *λ* windows determined using Gauss-Legendre quadrature nodes on [0, 1], where *λ* = 0 corresponds to full decoupling and *λ* = 1 to full coupling. Minimization and thermalization were performed at *λ* = 0.5 to provide geometries compatible with all windows. All bonds involving hydrogen atoms were constrained using the SHAKE algorithm (80), allowing a 2-fs integration timestep. A non-bonded cutoff of 10.0 Å was applied for van der Waals and short-range electrostatic interactions, and long-range electrostatic interactions were treated using the PME method (81). Production simulations of 5 ns per window (after 0.5-ns equilibration) were performed at 298 K using Langevin dynamics (86). Free energies were computed by numerically integrating ⟨∂*H*/∂*λ*⟩ using alchemlyb (87), with uncertainties estimated from four independent replicas. Experimental data were taken from the FreeSolv Database (Entries: mobley_20524 [Phenol], mobley_486214 [Syringol], mobley_3515580 [Guaiacol], mobley_3053621 [Benzene], mobley_2310185 [Ethanol], mobley_3259411 [Glycerol], mobley_7295828 [Anisole], mobley_8691603 [m-Cresol], mobley_3359593 [4-Propylguaiacol], mobley_7988076 [4-Hydroxybenzaldehyde], mobley_4683624 [4-Propylphenol]) (88).

### Simulation of lignin-solvation dynamics in water

Two 61-monomer softwood lignin polymers, L1a (one branch point) and L2 (two branch points), were obtained from previous work (89). Polymers were solvated in truncated octahedral TIP3P water boxes with a 14.0-Å buffer, yielding systems of ca. 92,000 atoms (L1a) and 58,000 atoms (L2), respectively. Each system was subjected to a three-stage energy minimization (5,000 steps each; 2,500 steepest descent followed by 2,500 conjugate gradient) with progressively reduced harmonic restraints on non-hydrogen lignin atoms (25.0, 15.0, 10.0 kcal mol^−1^ Å^−2^). The system was then heated from 100 to 300 K over 100 ps under NVT conditions with 10.0-kcal mol^−1^ Å^−2^ restraints applied. Subsequently, six 100-ps NPT equilibration stages were performed with restraints gradually reduced from 5.0 to 0.1 kcal mol^−1^ Å^−2^, followed by a final 100-ps unrestrained NPT equilibration. Production simulations were run under NPT conditions in successive 100-ns segments. Eight independent replicas per system were generated by assigning different random initial velocities. Covalent bonds involving hydrogen atoms were constrained using the SHAKE algorithm, permitting a 2-fs time step. Non-bonded interactions were truncated at 10.0 Å. Temperature was maintained at 300 K using the Langevin thermostat with a collision frequency of 1.0 ps^−1^, and pressure was regulated at 1 bar using the Berendsen barostat.

As part of the analysis of the trajectories, intramolecular and lignin-water hydrogen bonds were identified using distance (<3.5 Å) and angle (>135°) thresholds. First- and second-shell solvation water counts were obtained using cutoffs of 3.4 and 5.0 Å, respectively. π-π stacking contacts were identified by computing centroid-centroid distances and inter-normal angles for all aromatic ring pairs. Pairs with centroid distances <5.5 Å were classified as parallel (inter-normal angle <30°) or T-shaped (inter-normal angle 60°–90°). Water residence times were computed from the survival probability *P*(*t*), defined as the fraction of water molecules within 3.5 Å of any lignin heavy atom at time *t*_*0*_ that remain within this shell at time *t*_*0*_ + *t*. The residence time *τ* was extracted from a single-exponential fit of the form

(5) $$\begin{array}{c} P\left(t\right)=A\;\exp\left(-\frac{t}{\tau }\right) \end{array}\text{.}$$

If the single-exponential fit was poorly conditioned (amplitude *A* < 0.1), the fast-exchange component *τ*_1_ from a bi-exponential fit was used instead.

## Results & discussion

### Partial-charge assessment

**Figure 3:**
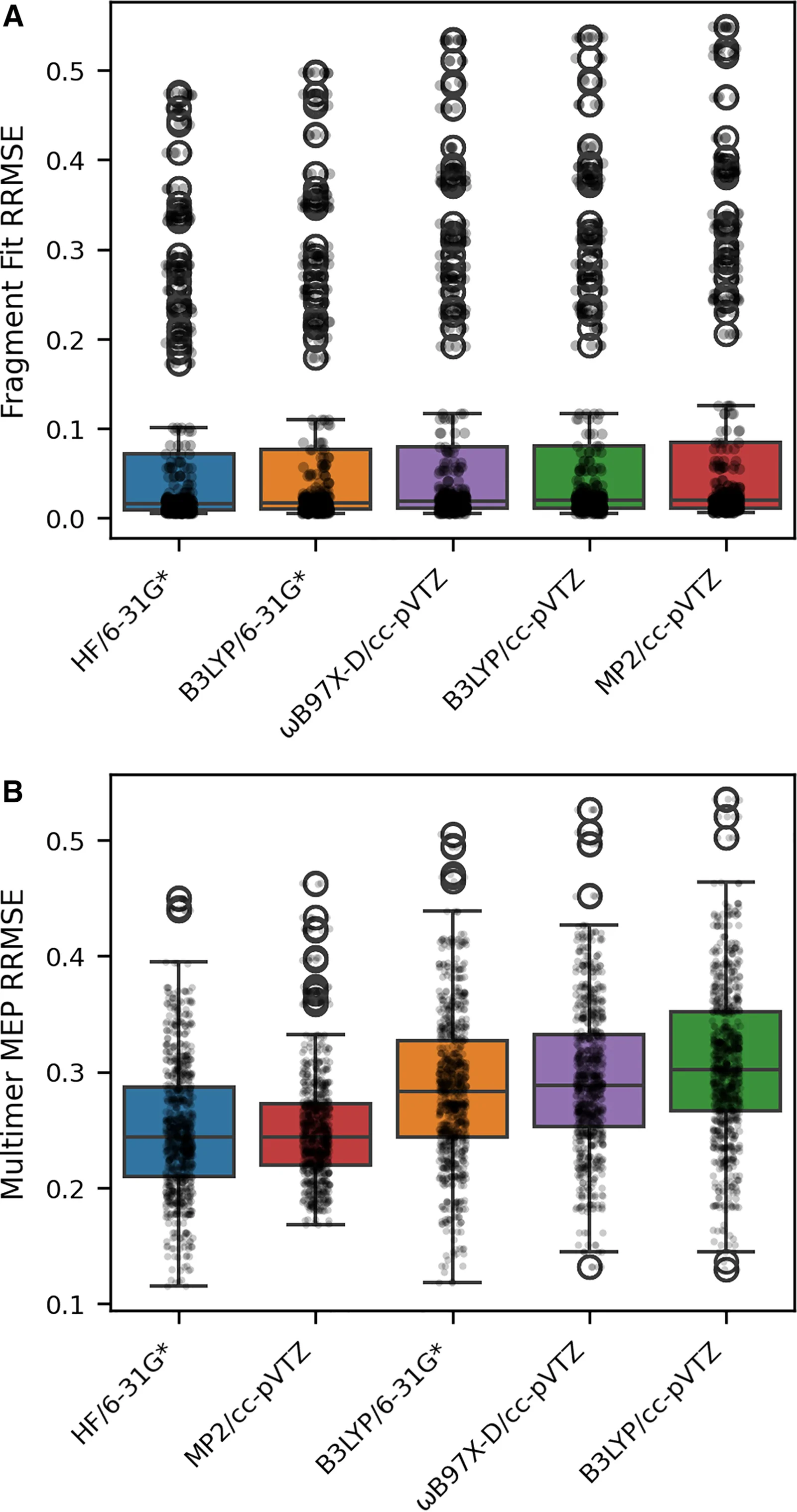
Quality of RESP charge fitting across theory levels (A) Distribution of fragment-level RRMSE of the comparison of MEP reproduced from RESP charge fits with quantum-mechanically calculated MEP at the respective level of theory. Each box represents the distribution across all fragments (*n* = 134) at the indicated theory level, with individual data points overlaid. (B) Distribution of MEP RRMSE for lignin multimers assembled from force-field fragments against quantum-mechanically calculated MEP of the multimer at the respective level of theory. Each box represents the distribution across the three most populated (Boltzmann-weighted) conformers per multimer (*n* = 200). Boxplots show median (*center line*), interquartile range (*box*), and 1.5× IQR (*whiskers*). Theory levels are ordered by mean value (best to worst, left to right). Combined data for charge restraint weights of 0.0003, 0.0005, 0.0007, and 0.001 are shown. Adjustment of charge restraint weights did not alter RRMSE distribution markedly (Fig. S4).

Since the LignAmb25 force field is founded upon GAFF2, all parameters except partial atomic charges could be used as a starting point. RESP fits on the capped force-field fragments were performed using MEPs calculated at five different levels of theory (HF/6-31G^∗^, B3LYP/6-31G^∗^, B3LYP/cc-pVTZ, ωB97X-D/cc-pVTZ, and MP2/cc-pVTZ), applying multiple charge restraint weights, to obtain partial atomic charges (Fig. 3). The quality of MEP reproduction, calculated at the same level of theory, was tested with regard to the RESP fits per fragment (Fig. 3 *A*), as well as for molecules assembled from RESP charge sets for fragments (Fig. 3 *B*). The comparison emphasized the robustness of RESP fits, as all levels of theory showed excellent results for most fragments with a mean RRMSE value of ca. 0.09 (Table S3). The similarity of fit results is further reflected in the distribution of outliers, which were nearly identical for all levels of theory tested. These outliers were identified as fragments with vicinal hydroxyl groups, which appear to create a MEP landscape that is difficult to capture using point charges. This hypothesis is further supported by the excellent correlation between fits with and without cap constraints, which showed *R*^2^ values ≥0.99 for all levels of theory (Fig. S2), suggesting that the additional constraints do not cause the outlier behavior.

Analysis of charge transferability to assembled molecules revealed that the density functional theory (DFT) methods overall performed worse than the (post-)HF methods, with the HF/6-31G^∗^ and MP2/cc-pVTZ levels of theory yielding the best results, and B3LYP/cc-pVTZ yielding the worst. The MEP of conjugated systems, such as hydroxystilbenes and systems containing cinnamate and benzoate linkages, was consistently found to be poorly reproduced for all investigated charge sets (Fig. S3).

One can expect to obtain overall elevated RRMSE values when applying fragment charges in the context of a larger molecule. However, the elevation may have also been influenced by the definition of the force-field fragment set, which was deliberately chosen to prioritize modularity over accuracy for single larger molecules by defining smaller, more general fragments. Consequently, this leads to disruption of conjugated systems, whose charge distributions cannot be properly captured when rebuilt from individual fragments. Furthermore, most capping groups on fragments were constrained to specific charges to ensure compatibility with GLYCAM, which removes further degrees of freedom.

### Optimization of bonded parameters

**Figure 4:**
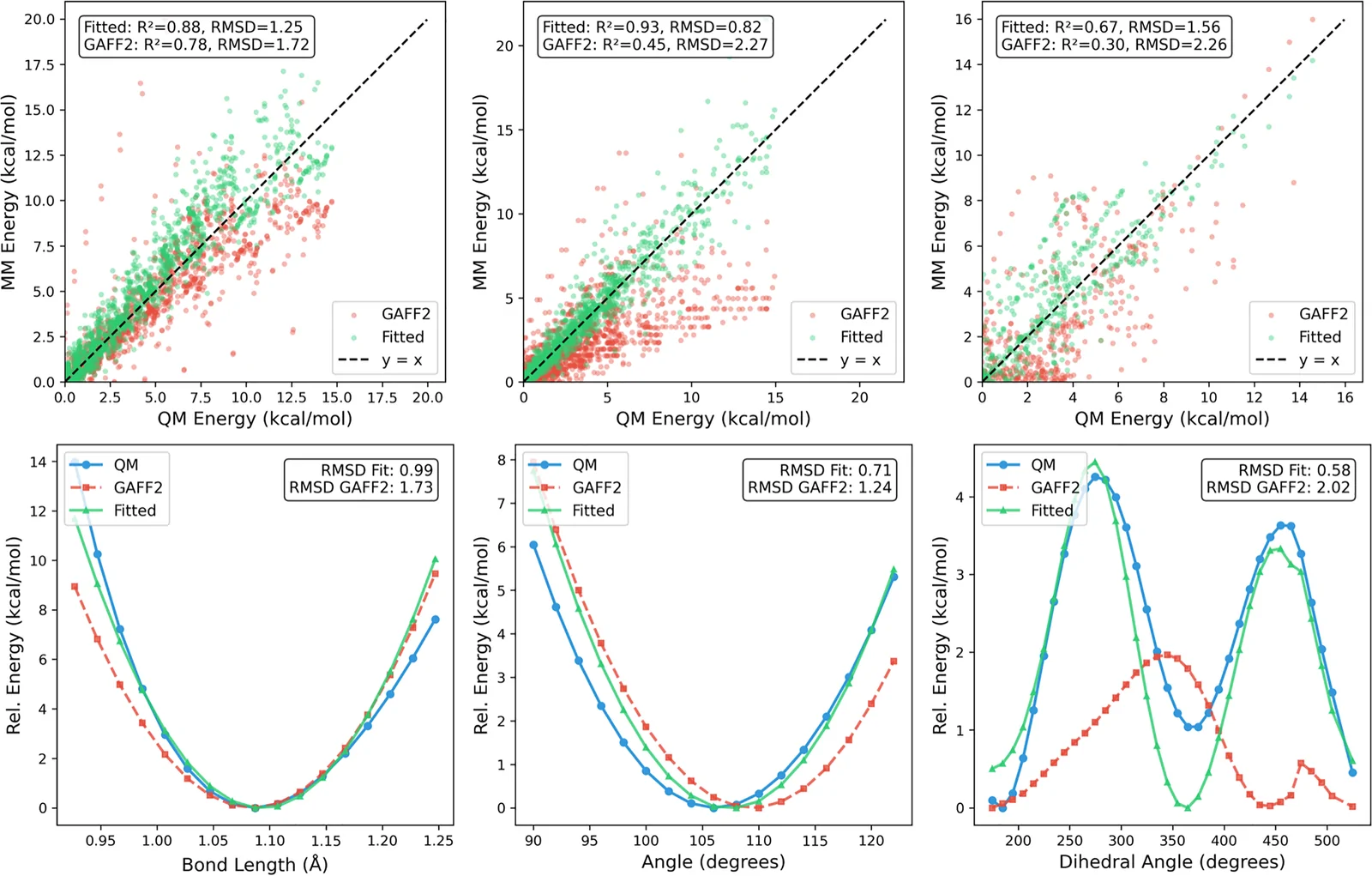
Comparison of fitted parameters of LignAmb25 with those from GAFF2 for bond, angle, and torsion terms at the B3LYP/cc-pVTZ level of theory Top row: parity plots comparing QM reference energies with MM energies for GAFF2 (*redf*) and fitted parameters (*green*) for bond-stretching, angle-bending, and torsion parameters, respectively. The dashed line represents perfect agreement (*y* = *x*). Fitted parameters show improved correlation with QM for bonds (*R*^2^ = 0.88 vs. 0.78), angles (*R*^2^ = 0.93 vs. 0.45), and torsions (*R*^2^ = 0.67 vs. 0.30). Bottom row: representative energy scans demonstrating improved agreement between fitted parameters and QM reference data: C–H bond stretch (*left*), H–O–C angle bend (*middle*), and C–C–O–H torsion rotation (*right*). In all cases, fitted parameters (*green*) reproduce the QM energy profile (*blue*) more closely than GAFF2 (*red*), with RMSD reductions of 43% (bond), 43% (angle), and 90% (torsion) for these examples.

Initial evaluation of a subset of GAFF2 parameters on lignin multimers revealed substantial deviation from PES data, which made reparametrization necessary (Fig. 4). RESP charge sets obtained with a charge restraint weight of 0.001 were used to optimize bonded parameters per level of theory, resulting in five candidate force fields (LignAmb25^HF/6-31G∗^, LignAmb25^MP2/cc-pVTZ^, LignAmb25^B3LYP/6-31G∗^, LignAmb25^B3LYP/cc-pVTZ^, and LignAmb25^ωB97X−D/cc-pVTZ^, named after the level of theory used for MEP calculation). The optimization protocol used PES scans of bonds, angles, and dihedrals for a set of model compounds covering various chemical environments present in lignin polymers (Fig. S5). A single conformer per compound was used for optimizing bond-stretching and angle-bending parameters, whereas a Boltzmann-weighted set of conformers was used to parametrize torsions. Marginal improvement in terms of reproducing bond-stretching energy profiles was achieved, as GAFF2 already covers this well. However, for angle-bending profiles, the fitting protocol achieved improved reproduction of high-energy regions, lowering the root-mean-square deviation (RMSD) across all profiles from 2.27 kcal mol^−1^ to 0.82 kcal mol^−1^. The high-energy regions are expected to be rarely sampled in simulations, but the improvement could be beneficial for dedicated sampling studies. The accuracy of torsion profile reproduction could be increased in low- and high-energy regions compared to GAFF2. The improvements also cover phase shifts and periodicity adjustments to match the QM-derived profiles (Fig. 4). Note, however, that the GAFF2 terms were purposely defined to work on a variety of organic compounds, which required the use of “generic” terms that may be less suitable for a dedicated compound class.

### Thermodynamic validation

**Figure 5:**
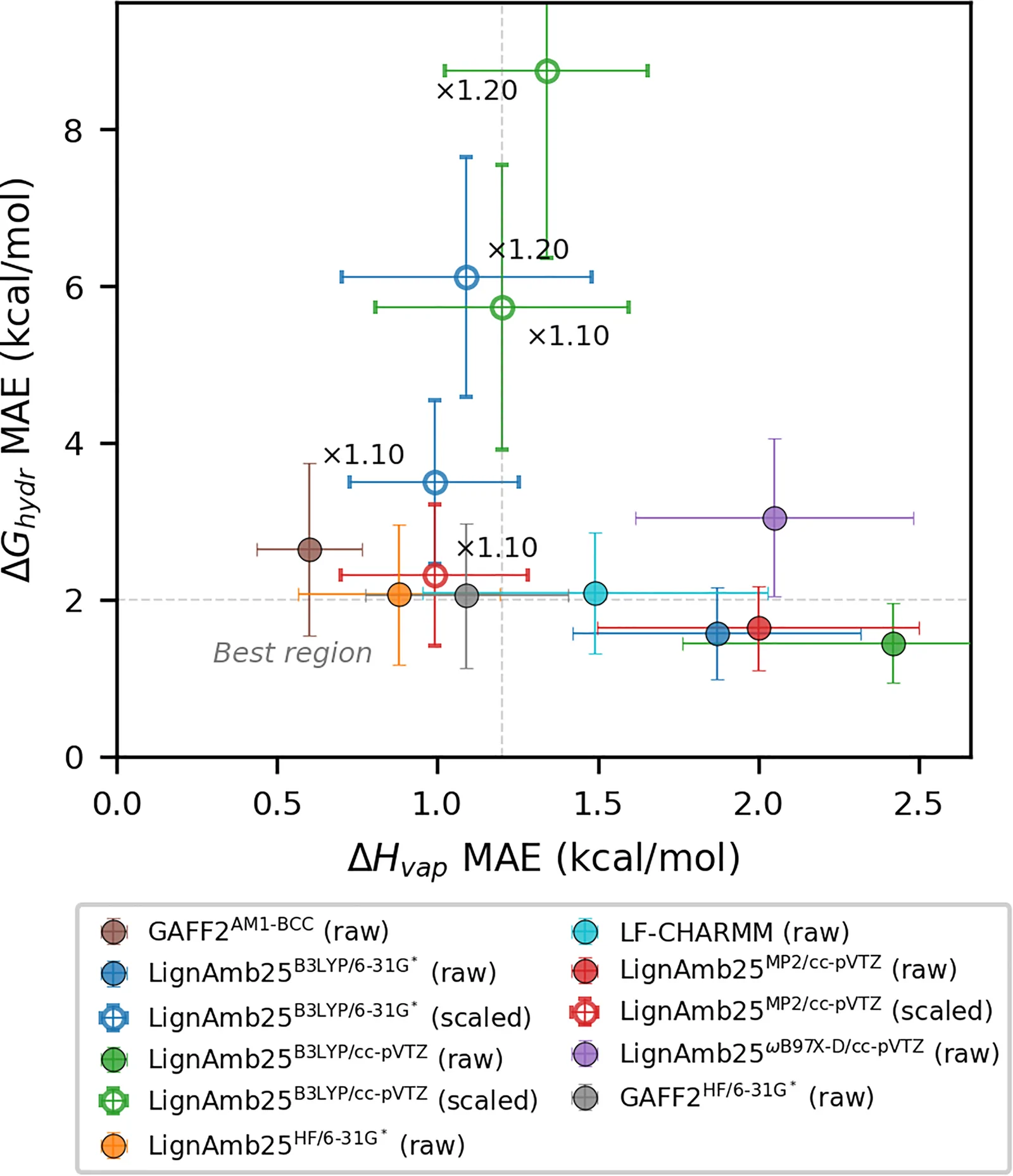
Trade-off between the prediction accuracy for heat of vaporization and hydration free energy Each point represents a charge model, with Δ*H*_vap_ MAE (*x* axis, *n* = 8 compounds) and Δ*G*_hydr_ MAE (*y* axis, *n* = 11 compounds). Filled circles indicate raw (unscaled) charges; open circles indicate scaled charges (×1.10 to ×1.20). Colors denote the QM theory level used for MEP calculation. Dashed lines indicate approximate thresholds for acceptable accuracy chosen based on published force-field benchmarks (90,91,92) (Δ*H*_vap_ MAE < 1.2 kcal mol^−1^, Δ*G*_hydr_ MAE < 2 kcal mol^−1^). Models in the lower-left region achieve good accuracy on both properties. Key models are labeled. Numerical data are available in the supporting material (Table S6; Table S7).

The optimized parameter sets per level of theory were used to compute enthalpies of vaporization (Δ*H*_vap_) and absolute hydration free energies (Δ*G*_hydr_) for a selection of lignin-related compounds (phenol, catechol, guaiacol, syringol, 4-hydroxybenzaldehyde, 4-propylguaiacol, 4-propylphenol) and small organic molecules that represent chemical environments found in lignin polymers (benzene, anisole, *m*-cresol, glycerol) to identify the best-performing parameter set based on Mean Absolute Error (MAE) against experimental values (Fig. 5).

The GAFF2 bonded parameter set with HF/6-31G^∗^ derived charges (GAFF2^HF/6-31G∗^) as well as AM1-BCC charges (GAFF2^AM1−BCC^) was used as a reference point. While GAFF2^AM1−BCC^ showed remarkable accuracy for reproduction of Δ*H*_vap_ with an MAE of 0.60 ± 0.74 kcal mol^−1^, it performed worse than GAFF2^HF/6-31G∗^ in reproducing absolute hydration free energy values with an MAE of 2.05 ± 0.92 kcal mol^−1^ compared to 2.64 ± 2.46 kcal mol^−1^ for GAFF2^HF/6-31G∗^. Considering that GAFF2^HF/6-31G∗^ also performed well in reproducing Δ*H*_vap_ values with an MAE of 1.10 ± 0.31 kcal mol^−1^, GAFF2^HF/6-31G∗^ arguably presents the more balanced reference force field. Convergence of the potential energies underlying the Δ*H*_vap_ and Δ*G*_hydr_ calculations was confirmed via block averaging (83), with propagated standard errors below 0.13 kcal mol^−1^ and 0.18 kcal mol^−1^, respectively, for all compounds and methods (Figs. S6 and S7). LignAmb25^HF/6-31G∗^ performed similarly, with MAE values 0.88 ± 0.31 kcal mol^−1^ and 2.06 ± 0.89 kcal mol^−1^. Interestingly, the LignAmb25^B3LYP/6-31G∗^, LignAmb25^B3LYP/cc-pVTZ^, and LignAmb25^MP2/cc-pVTZ^ parameter sets behaved very similarly in that they predict Δ*G*_hydr_ well but give worse estimates for Δ*H*_vap_. LignAmb25^B3LYP/cc-pVTZ^ yielded the best prediction of Δ*G*_hydr_ (MAE = 1.45 ± 0.51 kcal mol^−1^), but at the same time the worst Δ*H*_vap_ prediction (MAE = 2.42 ± 0.66 kcal mol^−1^). Simulations were also performed with parameters derived by following the parametrization scheme in the LF-CHARMM showcase (23). The CHARMM parameters were converted to the AMBER-compatible CHAMBER format and then simulated similarly to the other force fields under application of the CHARMM-modified TIP3P force field and force switching for Lennard-Jones interactions. The resulting parameter set yielded MAE values of 1.49 ± 1.93 kcal mol^−1^ and 2.08 ± 1.85 kcal mol^−1^ for Δ*H*_vap_ and Δ*G*_hydr,_ respectively, thus outperforming most of the tested parameter sets in terms of balancing the thermodynamic properties.

We experimented with rescaling B3LYP-derived charges by 1.10 and 1.20 to test whether the documented underestimation of dispersion effects in the B3LYP functional can be compensated for (93). Scaling of LignAmb25^B3LYP/cc-pVTZ^ charges by 1.10 considerably improved Δ*H*_vap_ prediction, lowering the MAE from 2.42 ± 0.66 kcal mol^−1^ to 1.20 ± 0.40 kcal mol^−1^ compared to the unscaled charges but also degraded Δ*G*_hydr_ prediction (MAE = 5.72 ± 1.81 kcal mol^−1^ vs. 1.45 ± 0.51 kcal mol^−1^obtained with unscaled charges). A similar effect was observed when rescaling LignAmb25^MP2/cc-pVTZ^ charges, which decreased the Δ*H*_vap_ MAE to 0.97 ± 0.30 kcal mol^−1^ (surpassing GAFF2^HF/6-31G∗^), but the Δ*G*_hydr_ MAE increased to 2.31 ± 1.03 kcal mol^−1^.

Ultimately, the unscaled LignAmb25^B3LYP/6-31G∗^, LignAmb25^B3LYP/cc-pVTZ^, and LignAmb25^MP2/cc-pVTZ^ parameter sets were selected for further testing as they seemingly provide superior hydration free energy estimates than the balanced alternatives (GAFF2^HF/6-31G∗^ and LignAmb25^HF/6-31G∗^). We decided this because lignin is a ubiquitous plant-derived, highly heterogeneous structural biopolymer that is typically processed and utilized in dissolved or dispersed form, which will most likely be simulated more often in explicit solvent rather than as a solid or liquid. Therefore, an accurate representation of solvent-solute interactions is crucial.

Note, however, that mixing of DFT- or MP2- with HF-derived charges from other biomolecular force fields, such as the ff19SB protein force field (31), could lead to unbalanced interaction energies. We account for this by providing two versions of LignAmb25: LignAmb25^HF^ (shorthand for LignAmb25^HF/6-31G∗^), which uses HF/6-31G^∗^-derived RESP charges to ensure compatibility with other AMBER force fields, and LignAmb25^Solo^, which, based on crystal simulation results highlighted in the section below, was chosen to be LignAmb25^MP2/cc-pVTZ^ and is meant for standalone use if lignin-solvation questions are in the focus. Both versions use a custom set of atom types as well as optimized bonded parameters obtained based on the respective charge set. Finally, note that the results in Fig. 5 are specific to the use of the TIP3P (85) water model. The prediction of thermodynamic properties using other popular water models, such as OPC (94), needs to be assessed in future work.

### Crystal structure simulations

**Figure 6:**
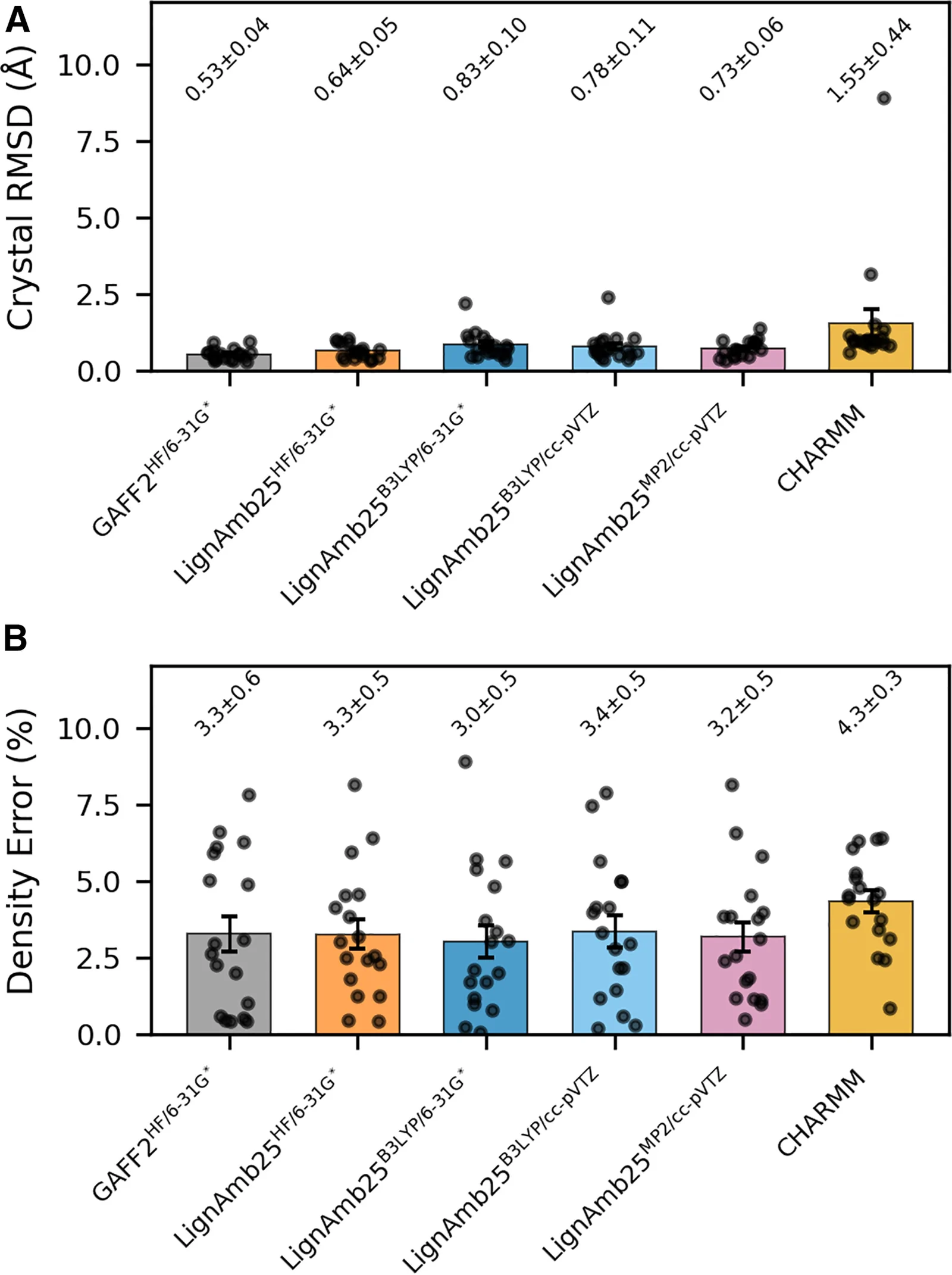
Crystal structure validation across force fields (A) Crystal heavy-atom RMSD and (B) mean absolute density error for 18 lignin-related crystal structures (Table S6) validated in both this work and the LF-CHARMM study (23). Data for GAFF2^HF/6-31G∗^ and all LignAmb25 force-field variants were obtained by taking the last 200 frames from the last 10 ns of a 20-ns-long MD simulation over four independent replicas. Bar heights represent mean values; error bars indicate standard error of the mean across structures. Density error calculated as |ρ_calc_/ρ_exp_ – 1| × 100%. Numerical data are available in the supporting material (Table S8).

As a final comparison, 18 lignin-related crystal structures (Table S6) previously simulated using LF-CHARMM (23)were simulated using the GAFF2^HF/6-31G∗^, LignAmb25^HF/6-31G∗^, LignAmb25^B3LYP/6-31G∗^, LignAmb25^B3LYP/cc-pVTZ^, and LignAmb25^MP2/cc-pVTZ^ force fields. RMSD of the unit cell against the experimental structures, as well as the density ratio of the experimental and simulated crystal structures, were computed and compared between the force fields (Fig. 6).

GAFF2^HF/6-31G∗^ performed best across all crystals with a mean density error of 3.3% ± 0.6% and a mean crystal RMSD of 0.53 ± 0.04 Å. All LignAmb25 force-field variants showed comparable performance in terms of mean density errors of ca. 3.0%, but varying performance regarding mean crystal RMSD. LignAmb25^B3LYP/cc-pVTZ^ and LignAmb25^B3LYP/6-31G∗^ showed RMSD values of 0.78 ± 0.11 and 0.83 ± 0.10 Å, respectively, and, in either case, at least one crystal melted. The LignAmb25^HF/6-31G∗^ set performed best out of the four LignAmb25 variants with a crystal RMSD of 0.64 ± 0.05 Å. LF-CHARMM also performed reasonably well, but worse than all LignAmb25 variants, with a mean density error of 4.3% ± 0.3% and a mean crystal RMSD of 1.55 ± 0.44 Å. It should be noted that the applied simulation protocol adheres to standard AMBER practices, which hinders comparability due to the application of harmonic restraints during equilibration of the systems. Therefore, simulations were repeated with a protocol faithful to the LF-CHARMM simulations, yielding results nearly identical to the AMBER-style simulations (Fig. S8).

The results for GAFF2^HF/6-31G∗^ underscore how well-behaved the GAFF2 parameter set is but also that the fitted parameters used in the LignAmb25 force-field variants yield results comparable to GAFF2^HF/6-31G∗^ for most crystal systems and even improve upon LF-CHARMM in many cases. Since LignAmb25^MP2/cc-pVTZ^ was found to be most consistent in retaining crystal packing, it is chosen as the final LignAmb25 parameter set meant for standalone use. Note that the systems with CSD accession codes INELIW and INELIW01 contain two additional dioxane molecules. It can therefore not be ruled out that the dioxane parameters (in either case obtained from GAFF2) contribute unfavorably to system stability.

### Solvation dynamics of lignin in aqueous solution

**Figure 7:**
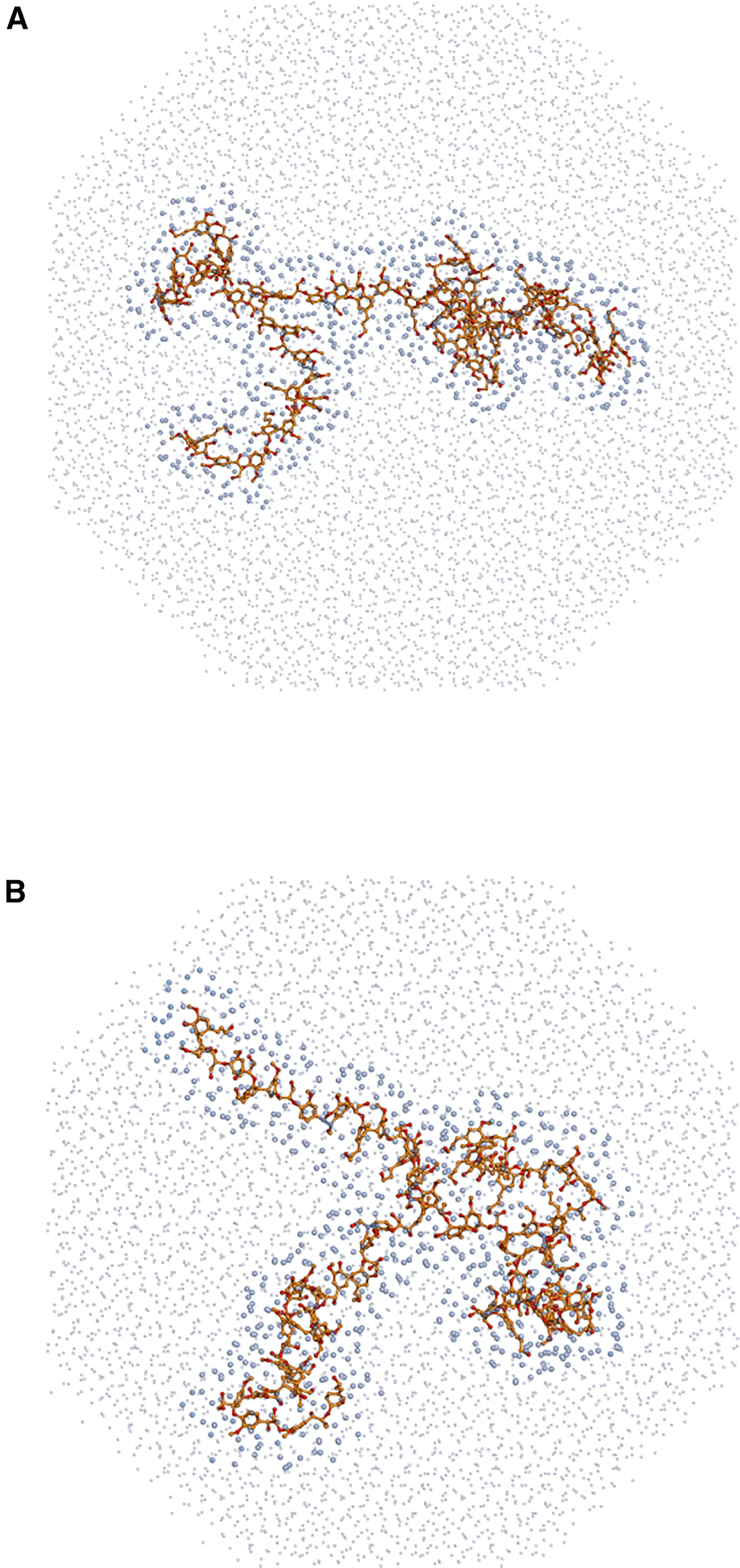
Systems used to study lignin-solvation dynamics in aqueous environment using MD simulations Polymer topologies represent common softwood lignin compositions and were taken from previously published work. (A) Lignin polymer with one branching point. (B) Lignin polymer with two branching points. Both polymers were solvated in TIP3P water boxes. Dots represent bulk water, and spheres represent water located in the solvation shell around the polymer.

**Figure 8:**
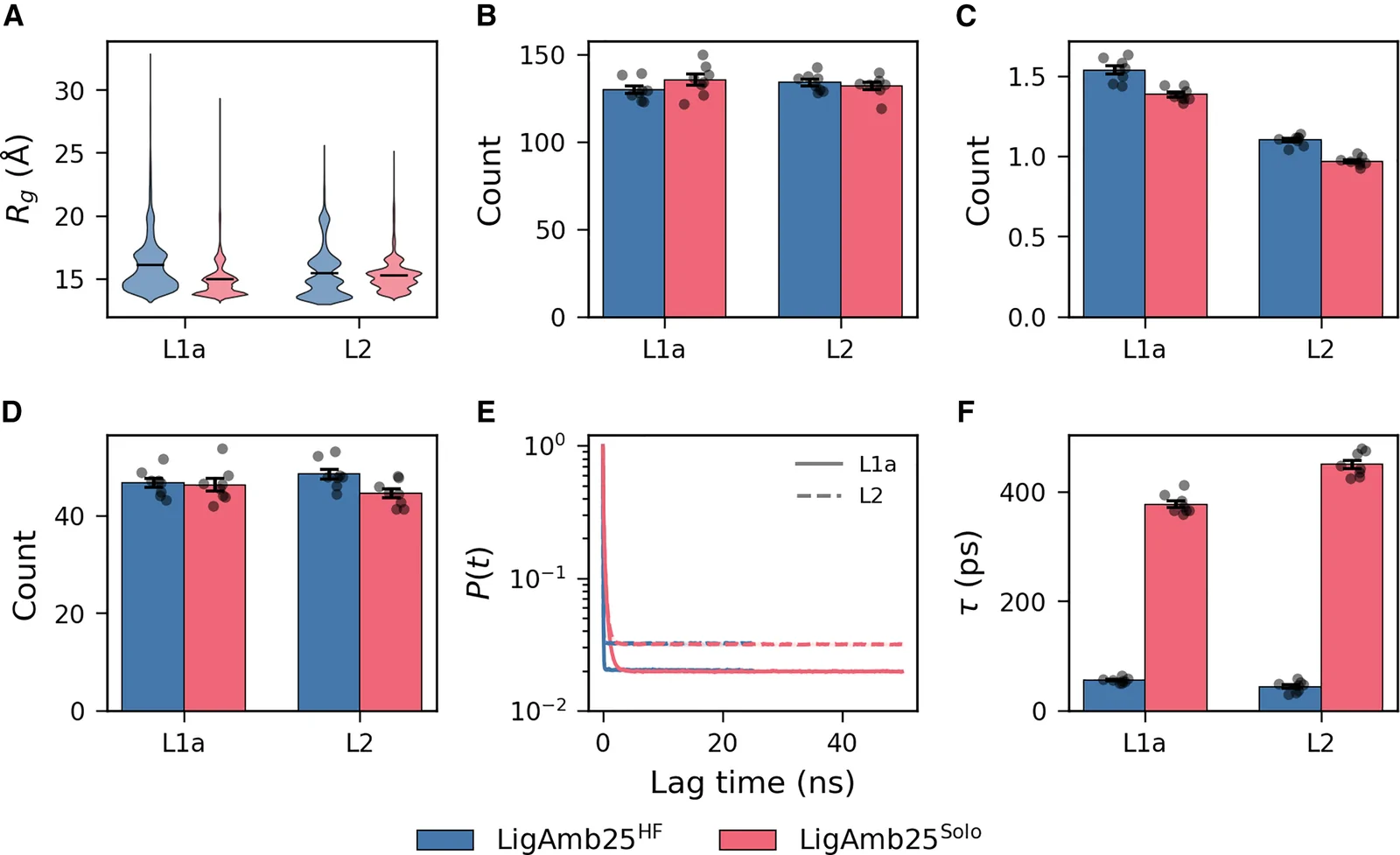
Comparison of structural and solvation properties of softwood lignin oligomers (L1a, one branch point; L2, two branch points; 61 G-units each) Data were obtained from 500-ns MD simulations in explicit TIP3P water over eight independent replicas; error bars denote standard error of the mean, with individual replicas shown as black dots. (A) Distribution of the radius of gyration *R*_*g*_. (B) Time-averaged number of intramolecular hydrogen bonds. (C) Time-averaged number of lignin-water hydrogen bonds. (D) Time-averaged number of π-π stacking contacts. (E) Water residence survival probability *P(t)* around the lignin surface (cutoff of 3.5 Å applied). (F) Water residence time *τ* extracted from exponential fits to *P(t)*.

To evaluate the practical consequence of charge-model choice on lignin-solvation behavior, we performed 500-ns MD simulations of two representative softwood lignin polymer models generated in prior work in explicit TIP3P water: model L1a (one branch point) and model L2 (two branch points) (Fig. 7) (89). Both polymers are composed of 61 G-units connected via a linkage composition typical for softwood lignin (50% β-O4, 30% 5-5, 10% α-O4, 10% β-5). Both polymers were simulated using the LignAmb25^Solo^ and LignAmb25^HF^ force fields and analyzed with respect to the radius of gyration (R_g_), intra- and intermolecular hydrogen-bonding patterns, π-π stacking contacts, and water residence time (Fig. 8).

As shown in Fig. 8, *A*, both force fields yield compact, globular conformations with comparable radii of gyration of around 15 Å. Furthermore, the number of registered intramolecular hydrogen bonds (Fig. 8 *B*) and π-π stacking contacts (Fig. 8 *D*) is very similar between the two force fields. The number of direct lignin-water hydrogen bonds is low, consistent with a collapsed polymer that largely excludes solvent from its interior (Fig. 8 *C*). Despite the structural similarity, the two charge models produce remarkably different solvation dynamics. The water residence survival probability *P*(*t*) decays substantially faster with the universal LignAmb25^HF^ compared to the solvent-optimized LignAmb25^Solo^, and the corresponding residence times differ by nearly an order of magnitude (*τ* ca. 50 ps vs. *τ* ca. 400 ps; Figs. 8, *E* and *F*). This indicates that the solvent-optimized charges sustain a more persistent hydration shell around the lignin surface, likely due to stronger electrostatic lignin-water interactions. These results demonstrate that, while both parameterizations produce qualitatively similar equilibrium structures in aqueous solution, the choice of a charge model has a pronounced effect on the dynamics of the solvation shell and thus on properties driven by lignin-solvent interactions such as solubility, aggregation kinetics, and thermodynamics of lignin in mixed-solvent systems.

### Conclusions

We have developed LignAmb25, a molecular mechanics force field for lignin and lignin-related compounds with systematically derived partial charges that is available as both a standalone (LignAmb25^Solo^) and AMBER-compatible (LignAmb25^HF^) version. LignAmb25 contains parameters for all common monolignol units and their associated linkages, along with less commonly encountered units such as tricin, spirodienones, and hydroxystilbenes. Through comprehensive evaluation of RESP charge fitting across five QM theory levels, we identified B3LYP/cc-pVTZ as the best method for generating transferable charges that balance MEP reproduction with thermodynamic and structural accuracy. The chosen validation strategy revealed that MEP reproduction alone is an insufficient criterion for charge-model selection. While all theory levels achieved good fragment-level fits (RRMSE <0.10), thermodynamic validation exposed significant differences in predictive accuracy.

The LignAmb25^Solo^ parameter set yielded a MAE of 2.00 ± 0.50 kcal mol^−1^ for enthalpy of vaporization calculations and hydration free energies with acceptable accuracy (1.64 ± 0.54 kcal mol^−1^) for the lignin-related compounds tested, while MAE values of 0.88 ± 0.31 kcal mol^−1^, and 2.06 ± 0.89 kcal mol^−1^ for enthalpy of vaporization, and hydration free energy calculations, respectively, were obtained using the LignAmb25^HF^ parameter set. Comparison with existing force fields demonstrates that LignAmb25 provides competitive performance. Crystal structure simulations on 18 structures common to both this work and the one describing the LF-CHARMM showed comparable density reproduction across the force fields GAFF2^HF/6-31G∗^ (3.3% ± 0.6%), LignAmb25^HF^ (3.3% ± 0.5%), LignAmb25^Solo^ (3.2% ± 0.5%), and LF-CHARMM (4.3% ± 0.3%). GAFF2^HF/6-31G∗^ achieved the lowest crystal RMSD (0.53 ± 0.04 Å), followed by LignAmb25^HF^ (0.64 ± 0.05 Å), LignAmb25^Solo^ (0.73 ± 0.06 Å), and LF-CHARMM (1.55 ± 0.44 Å), although the LF-CHARMM results include one structure that exhibited crystal melting. Although GAFF2^HF/6-31G∗^ showed remarkable performance, LignAmb25^HF^ delivered comparable results and was optimized to reproduce PES profiles more accurately in high-energy regions, which may be beneficial for extensive sampling studies. To further probe the practical consequences of the charge-model choice in a condensed-phase setting directly relevant to lignin applications, we performed explicit-solvent MD simulations of two 61-monomer softwood lignin oligomers solvated in TIP3P water at 300 K using both the LignAmb25^Solo^ and LignAmb25^HF^ force fields. Both parameter sets produced compact, globular conformations with comparable radii of gyration (ca. 15 Å), intramolecular hydrogen-bond networks, and π-π stacking contacts, indicating that the equilibrium structure of lignin in water is largely insensitive to the charge model. However, the solvation dynamics differed markedly, as the water residence time around the lignin surface is approximately 8-fold shorter with LignAmb25^HF^ (*τ* ca. 50 ps) than with LignAmb25^Solo^ (*τ* ca. 400 ps), reflecting the stronger electrostatic lignin-water interactions encoded in the solvent-optimized charges. This demonstrates that, while both parameterizations are suitable for studying lignin conformational properties, the choice of charge model may be consequential for properties governed by lignin-solvent interactions, such as solubility predictions or aggregation kinetics.

Atom types and residue names in the LignAmb25 force field were carefully chosen to ensure compatibility with the major biomolecular force fields and GAFF2, allowing seamless integration into the AMBER MD suite. We acknowledge that the large number of fragments in the force field and their unintuitive naming make structure preparation tedious. Thus, we will focus on developing a tool for automated preparation of lignin simulation systems, similar to what pdb4amber in AmberTools (24) provides for proteins. A prototype of this was used in this study to prepare most of the simulation systems.

Future work will focus on curing the imbalance between Δ*H*_vap_ and Δ*G*_hydr_ by exploiting tools such as ForceBalance to optimize Lennard-Jones parameters and possibly also charge parameters. Additionally, reparametrization of the LignAmb25^Solo^ charges, and consequently the torsion parameters, without the GLYCAM-compatible charge constraints must be explored to determine changes in the reproduction of condensed-phase properties of lignin-related compounds. Furthermore, the QM data collected in this work represent the foundation for future machine-learning potential (MLP) development. However, development of a robust MLP would require further extension of the dataset and inclusion of intermolecular configuration sampling. This marks the natural next step for a data-driven description of lignin and can be directly compared to the classical parameter set developed in this work. Finally, LignAmb25 is the most comprehensive lignin force field available to date, but it is by no means complete and could be extended in the future to include additional caps (e.g., methyl caps on hydroxyl groups) or tails (e.g., aminated tails) to capture the chemical diversity of lignin even more thoroughly and understand the role of these modifications better. This also concerns the modeling of lignin-protein crosslinks, which are yet to be detected *in vivo*, but have been found to be chemically feasible (95). The modular approach of LignAmb25 makes adding new residues to address these topics straightforward. The LignAmb25 parameter and library files will be distributed with AMBER starting with AMBER26.

## Data and code availability

The LignAmb25 parameter and library files are available within the Amber suite of biomolecular simulation programs as of AMBER26. The Amber suite is available at https://ambermd.org. Input files and analysis scripts for charge derivation and for computations of enthalpy of vaporization, absolute hydration free energy, and crystal simulations have been deposited at https://researchdata.hhu.de/ (https://doi.org/10.25838/d5p-92) and are publicly available.

## Acknowledgments

This work was funded by a grant from the Ministry of Innovation, Science, and Research of North-Rhine Westfalia (NRW) within the framework of the NRW Strategieprojekt BioSC (no. 313/323-400-002 13) by the BOOST FUND 2.0 project “OptiCellu” and, in part, by the 10.13039/501100001659Deutsche Forschungsgemeinschaft (DFG, German Research Foundation) grant 270650915 (Research Training Group GRK 2158). We are grateful for the computational infrastructure and support provided by the “Zentrum für Informations- und Medientechnologie” (ZIM) at Heinrich Heine University Düsseldorf and the computing time provided by the John von Neumann Institute for Computing (10.13039/100013137NIC) to H.G. on the supercomputer JUWELS at Jülich Supercomputing Centre (JSC) (user IDs: VSK33, lignin). We acknowledge the developers of AMBER and the RDKit cheminformatics package for making their software publicly available.

## Author contributions

H.G. designed the research. M.L. derived the force-field parameters and performed validation. M.B. assisted in the modeling of electrostatic interactions and torsion parameters. J.G. assisted in choosing suitable methods for exploring conformational space. M.L., M.B., and H.G. wrote the manuscript. H.G. secured funding.

## Declaration of interests

The authors declare no competing interests.

## Supporting material

Document S1. Figures S1–S8 and Tables S1–S8

Document S2. Article plus Supporting material
