# Supplementary material for: LignAmb25: A comprehensive AMBER force field addressing lignin’s structural and chemical diversity
Source: Biophys J. 2026 Apr 7;125(16):4391–406. doi: 10.1016/j.bpj.2026.04.004 (PMC13507438; doi:10.1016/j.bpj.2026.04.004)
Supplement: Supplementary file 1 [file mmc1.pdf]

Biophysical Journal, Volume *125*

Supplemental information

LignAmb25: A comprehensive AMBER force field addressing lignin's structural and chemical diversity

Marco Lapsien, Michele Bonus, Julian Greb, and Holger Gohlke

Biophysical Journal, Volume *125*

Supplemental information

LignAmb25: A comprehensive AMBER force field addressing lignin's structural and chemical diversity

Marco Lapsien, Michele Bonus, Julian Greb, and Holger Gohlke

LignAmb25: A Comprehensive AMBER Force Field Addressing Lignin's Structural and Chemical Diversity

Marco Lapsien[1], Michele Bonus[1], Julian Greb[1] and Holger Gohlke[1,2,3*]

[1]Institute for Pharmaceutical and Medicinal Chemistry, Heinrich Heine University Düsseldorf, 40225 Düsseldorf, Germany

[2]Bioeconomy Science Center (BioSC), Heinrich Heine University Düsseldorf, 40225 Düsseldorf, Germany

[3]Institute of Bio- and Geosciences (IBG-4: Bioinformatics), Forschungszentrum Jülich, 52425 Jülich, Germany

*Address: Universitätsstr. 1, 40225 Düsseldorf, Germany.

Phone: (+49) 211 81 13662

E-mail: gohlke@uni-duesseldorf.de

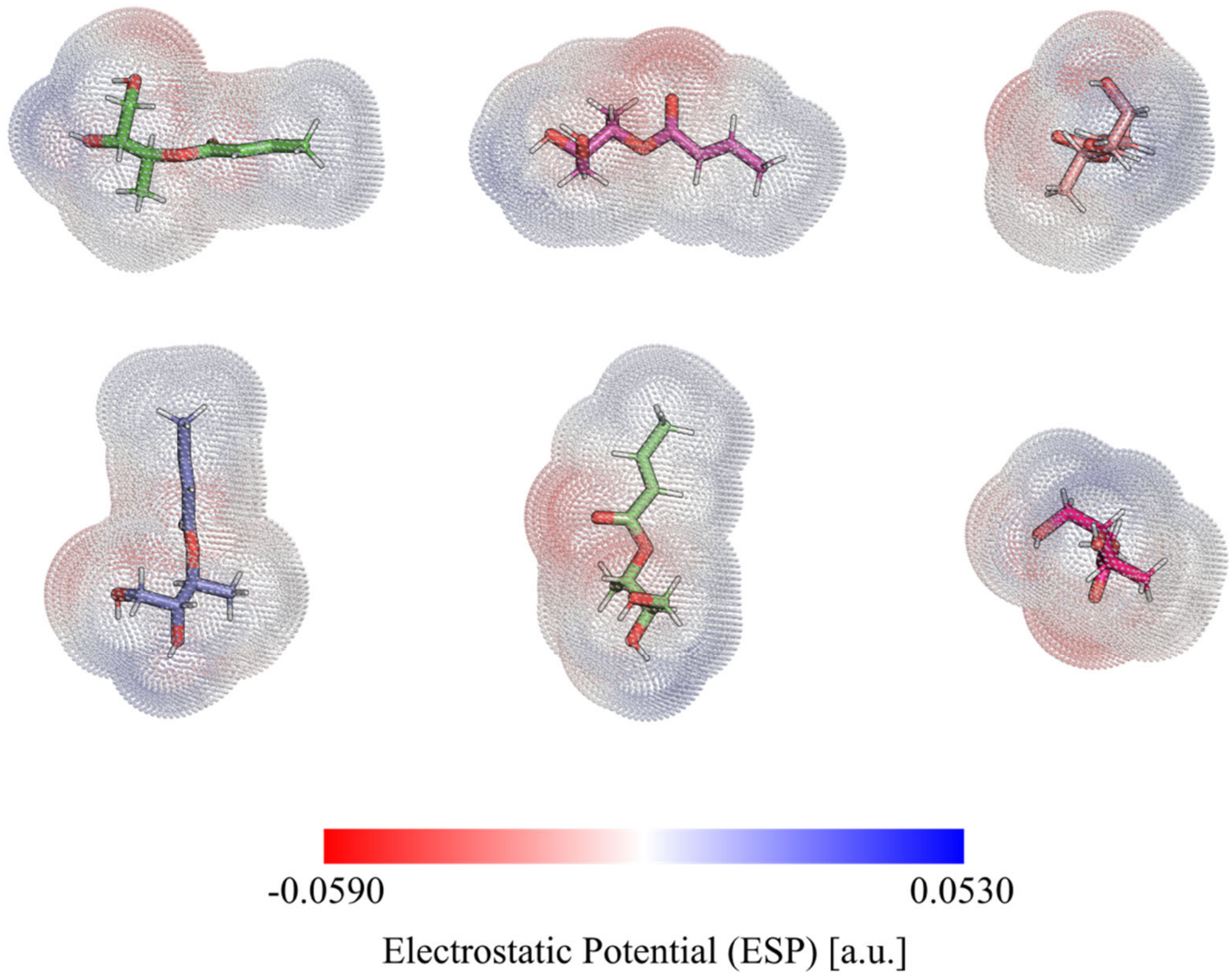

Figure S1: Example for orientation sampling performed for RESP Fitting. Orientations from top left to bottom right: Initial orientation, 90° rotation around x-axis, 90° rotation around y-axis, 90° rotation around z-axis, 90° rotation around x-axis + 90° rotation around z-axis, 90° rotation around y-axis + 90° rotation around z-axis.

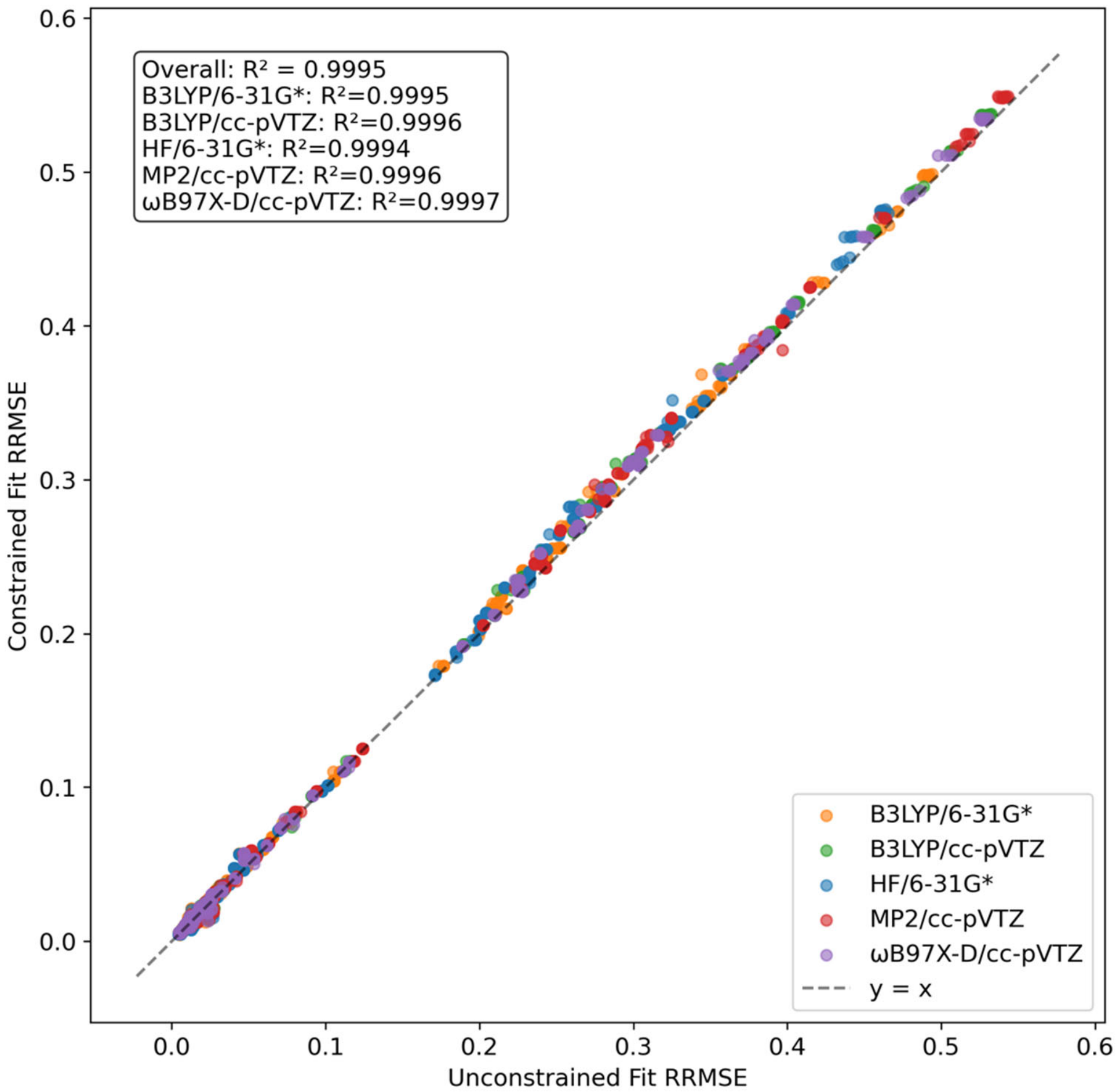

Figure S2: Correlation between RRMSE of RESP Fits with and without cap constraints.

Combined results over all RESP charge restraint weights per level of theory studied are shown.

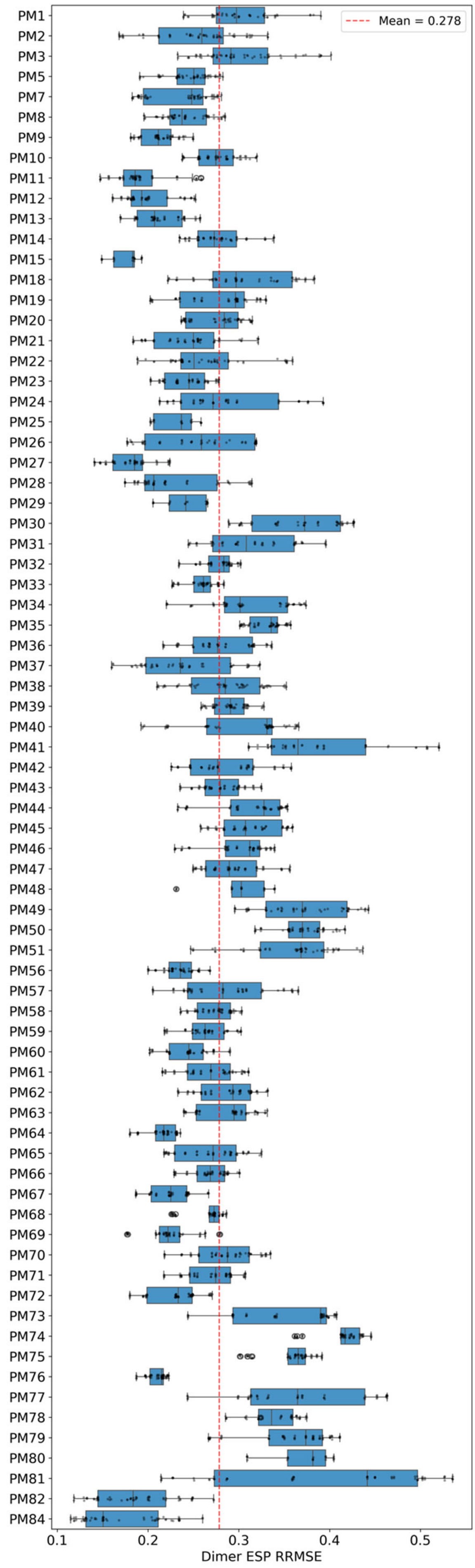

Mean = 0.278
PM1
PM2
PM3
PM5
PM7
PM8
PM9
PM10
PM11
PM12
PM13
PM14
PM15
PM18
PM19
PM20
PM21
PM22
PM23
PM24
PM25
PM26
PM27
PM28
PM29
PM30
PM31
PM32
PM33
PM34
PM35
PM36
PM37
PM38
PM39
PM40
PM41
PM42
PM43
PM44
PM45
PM46
PM47
PM48
PM49
PM50
PM51
PM56
PM57
PM58
PM59
PM60
PM61
PM62
PM63
PM64
PM65
PM66
PM67
PM68
PM69
PM70
PM71
PM72
PM73
PM74
PM75
PM76
PM77
PM78
PM79
PM80
PM81
PM82
PM84
0.1
0.2
0.3
0.4
0.5
Dimer ESP RRMSE

Figure S3: RRMSE distribution of ESP reproduction of lignin multimers based on force field fragment RESP charges. Data was taken for each analyzed charge restraint weight of all levels of theory studied.

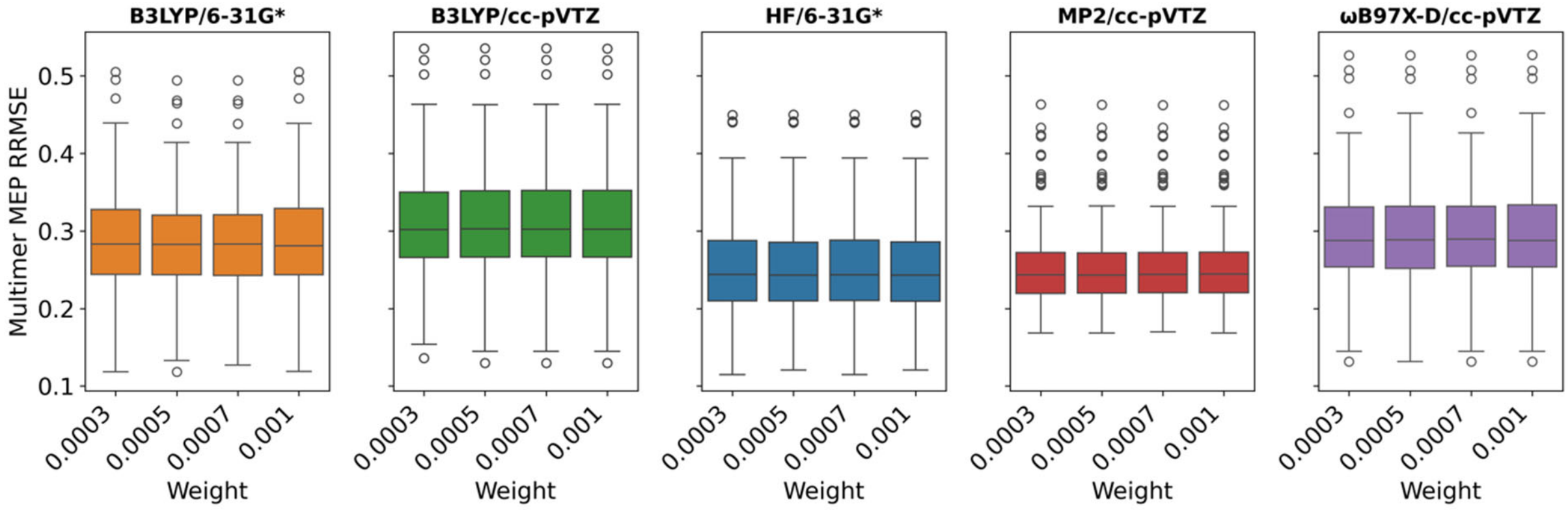

Figure S4: Comparison of ESP reproduction for dimers from RESP charges for different charge restraint weights and levels of theory.

Interunit linkages

A

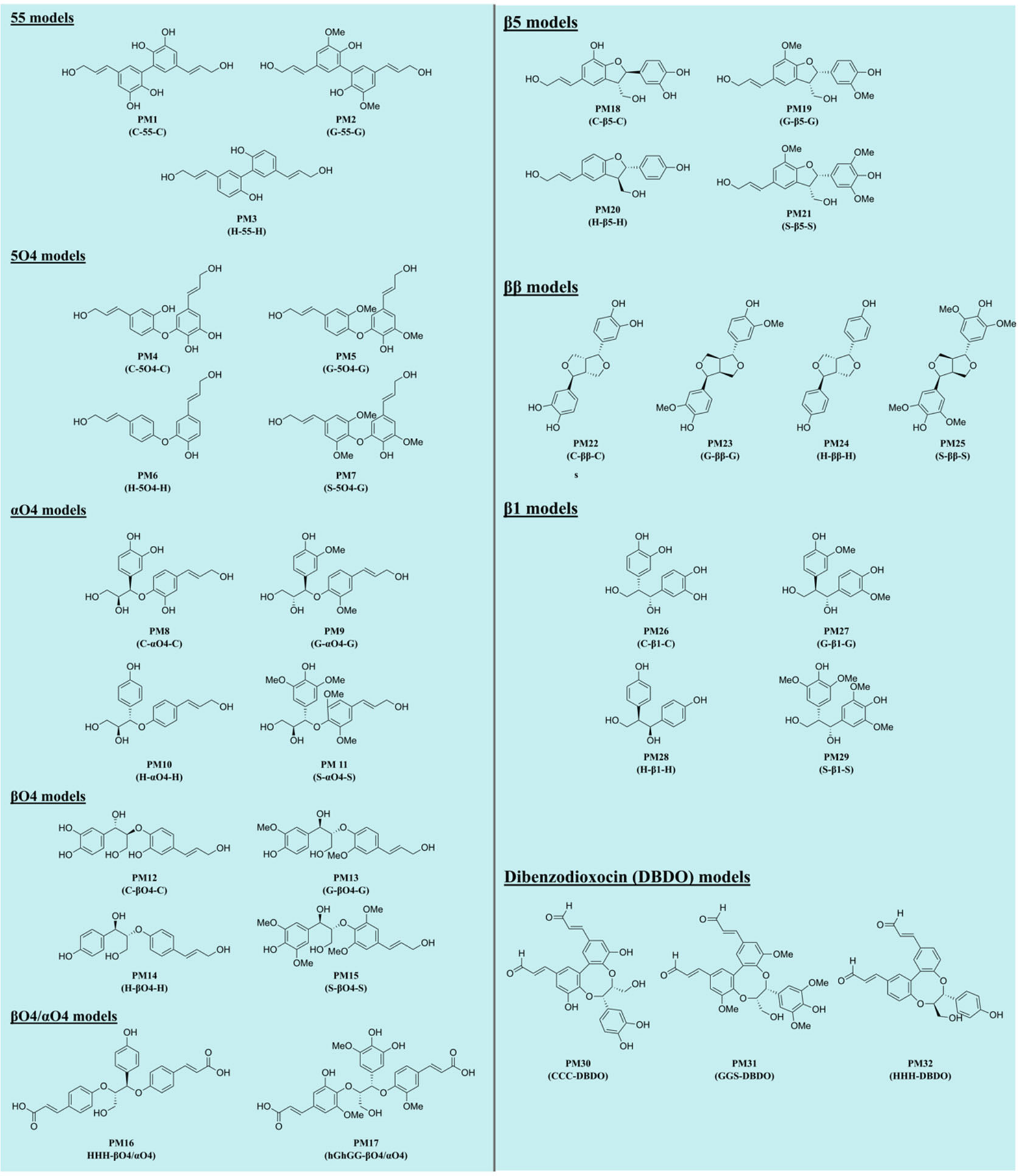

Hydroxycinnamic acid conjugates

B

α-Cinnamate (αCM) models

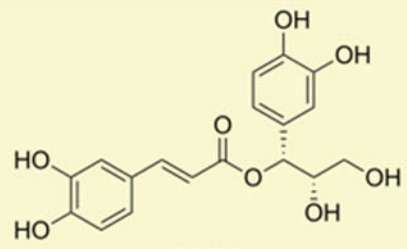

PM33
(C-αCM-C)

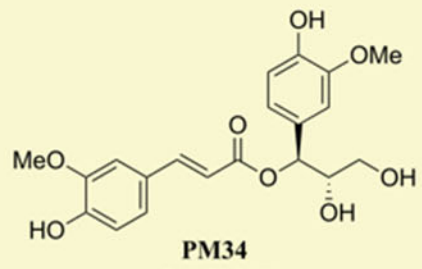

PM34
(G-αCM-G)

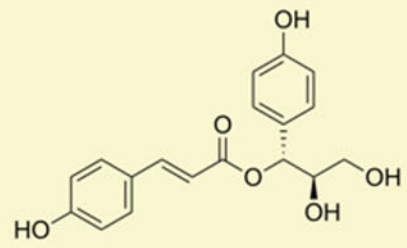

PM35
(H-αCM-H)

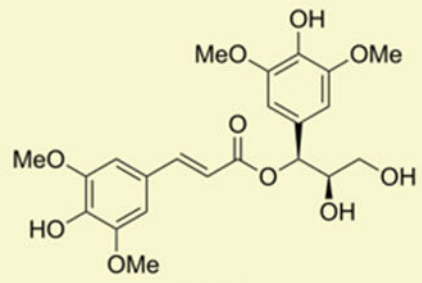

PM36
(S-αCM-S)

γ-Cinnamate (γCM) models

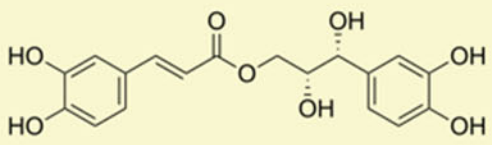

PM37
(C-γCM-C)

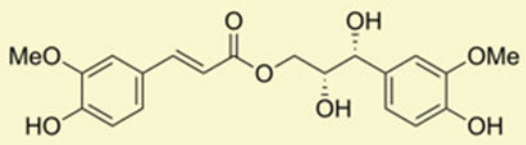

PM38
(G-γCM-G)

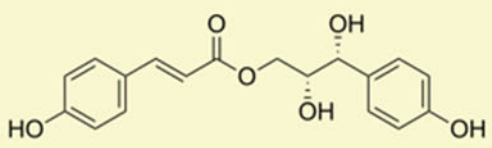

PM39
(H-γCM-H)

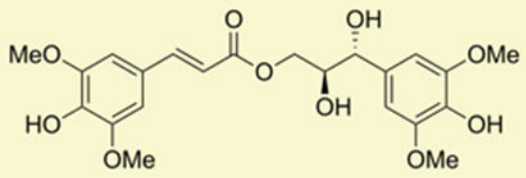

PM40
(S-γCM-S)

α-Benzoate (αBZ) models

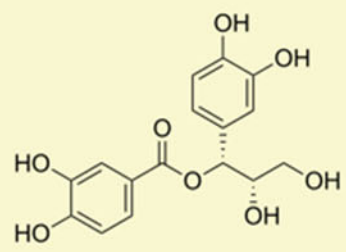

PM41
(C-αBZ-C)

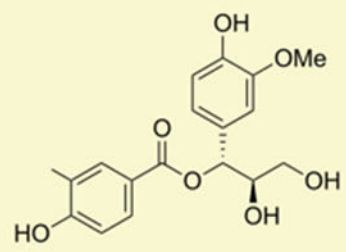

PM42
(G-αBZ-G)

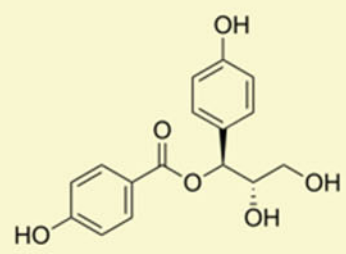

PM43
(H-αBZ-H)

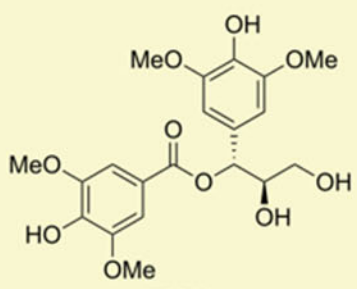

PM44
(S-αBZ-S)

γ-Benzoate (γBZ) models

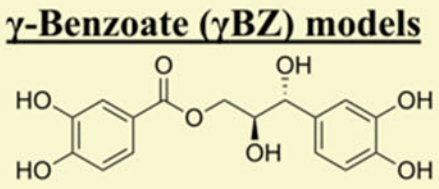

PM45
(C-γBZ-C)

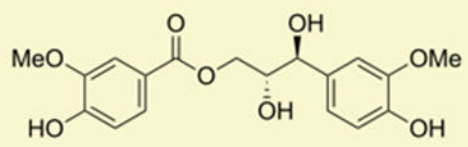

PM46
(G-γBZ-G)

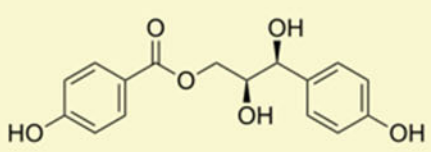

PM47
(H-γBZ-H)

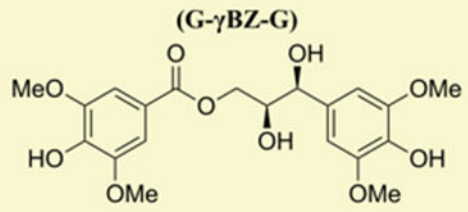

PM48
(S-γBZ-S)

Diferulate (DF) models

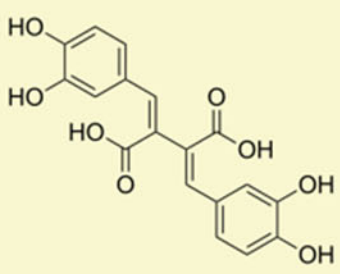

PM49
(C-DF-C)

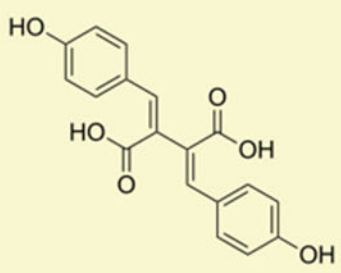

PM50
(H-DF-H)

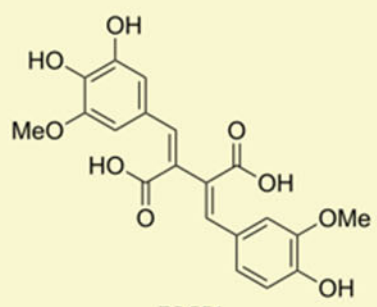

PM51
(hG-DF-G)

Cyclic diferulate (cycDF) models

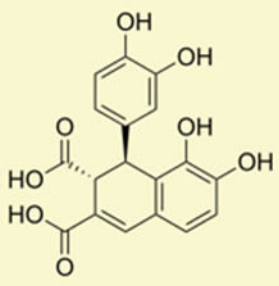

PM52
(C-cycDF-C)

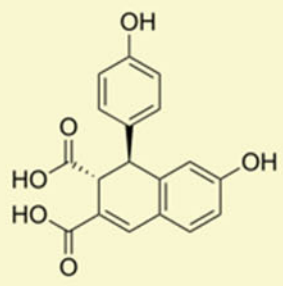

PM53
(H-cycDF-H)

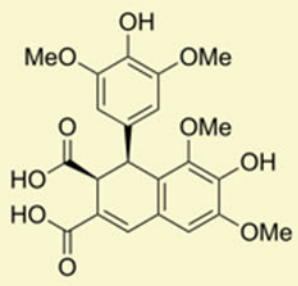

PM54
(S-cycDF-S)

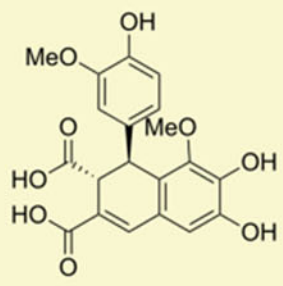

PM55
(hG-cycDF-G)

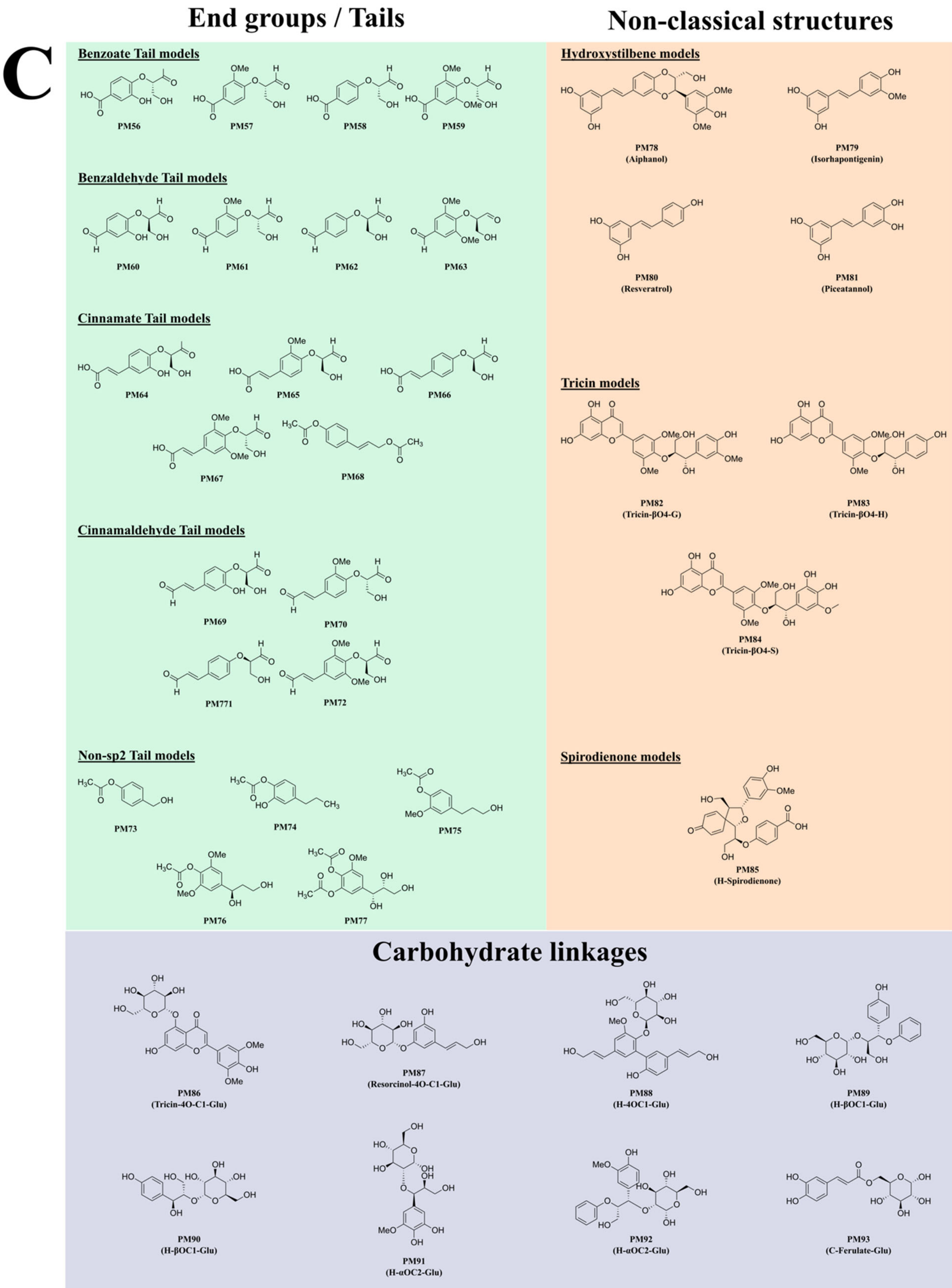
End groups / Tails
Non-classical structures
C
Benzoate Tail models
PM56
PM57
PM58
PM59
Benzaldehyde Tail models
PM60
PM61
PM62
PM63
Cinnamate Tail models
PM64
PM65
PM66
PM67
PM68
Cinnamaldehyde Tail models
PM69
PM70
PM771
PM72
Non-sp2 Tail models
PM73
PM74
PM75
PM76
PM77
Hydroxystilbene models
PM78 (Aiphanol)
PM79 (Isorhapontigenin)
PM80 (Resveratrol)
PM81 (Piceatannol)
Tricin models
PM82 (Tricin-βO4-G)
PM83 (Tricin-βO4-H)
PM84 (Tricin-βO4-S)
Spirodienone models
PM85 (H-Spirodienone)
Carbohydrate linkages
PM86 (Tricin-4O-C1-Glu)
PM87 (Resorcinol-4O-C1-Glu)
PM88 (H-4OC1-Glu)
PM89 (H-βOC1-Glu)
PM90 (H-βOC1-Glu)
PM91 (H-αOC2-Glu)
PM92 (H-αOC2-Glu)
PM93 (C-Ferulate-Glu)

Figure S5: Parameterization models covering end groups, carbohydrate linkages, and less commonly encountered lignin monomers. A: Models covering commonly encountered lignin linkages. B: Models covering hydroxycinnamic acid conjugates encountered in lignin polymers. C: Models covering end groups, carbohydrate linkages, and less commonly encountered lignin monomers. All models except for lignin-carbohydrate models were used for charge transferability assessment due to GLYCAM charges being predetermined in the latter case.

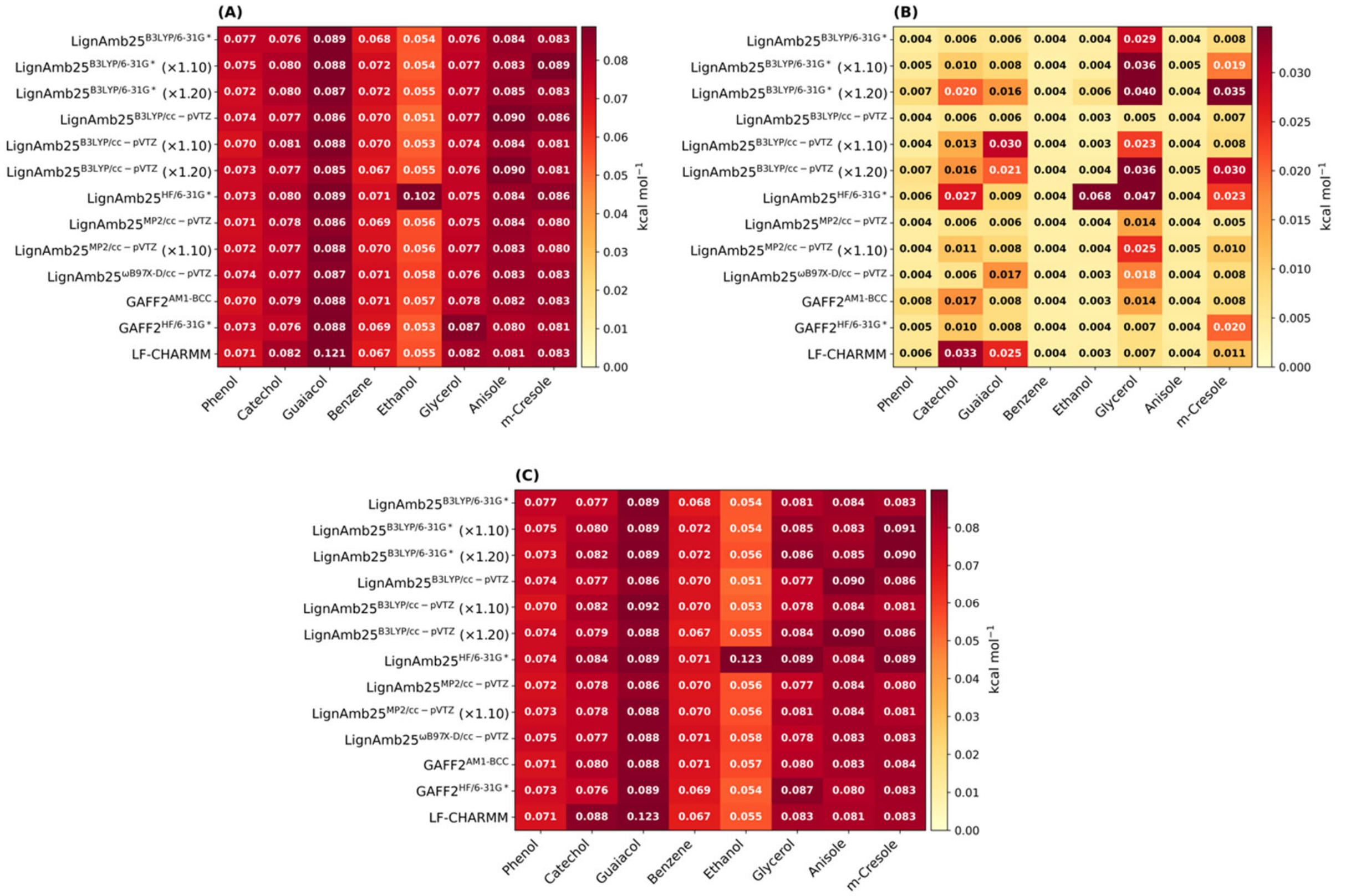

Figure S6: Block-averaged standard errors of the mean (SEM) for (A) gas-phase potential energy U_g, (B) liquid-phase potential energy per molecule U_l, and (C) the enthalpy of vaporization ΔH_{vap}, obtained via the Flyvbjerg & Petersen blocking transformation. Values for ΔH_{vap} were propagated as $\mathbf{SEM}(\boldsymbol{\Delta H_{vap}}) = \sqrt{(\mathbf{SEM}(\boldsymbol{U_g})^2 + \mathbf{SEM}(\boldsymbol{U_l})^2)}$ Each entry represents the root-mean-square of the converged per-replica SEMs across four independent replicas. All propagated ΔH_{vap} uncertainties are below 0.13 kcal mol⁻¹, confirming adequate convergence of the 10 ns production simulations.

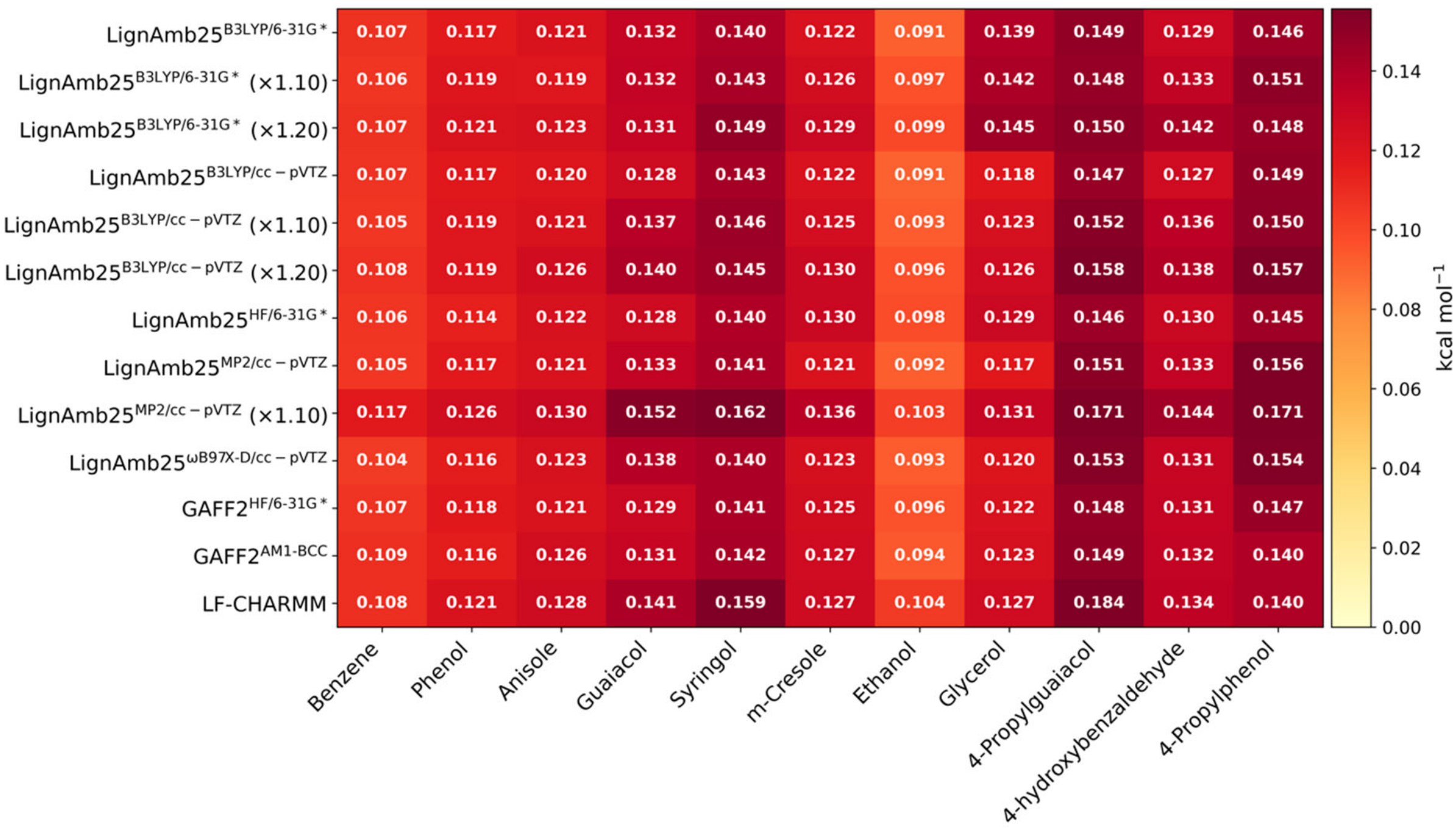

Figure S7: Block-averaged standard errors of the mean (SEM) for the hydration free energy ΔG_{hydr}, obtained via the Flyvbjerg & Petersen blocking transformation. Per-lambda SEMs were propagated through trapezoidal integration as $\boldsymbol{SEM(\Delta G_{hydr}) = \sqrt{\sum w_i^2 \cdot SEM_i^2}}$, where w_i are the trapezoid-rule weights for the 1-point Gauss–Legendre quadrature. Each entry represents the root-mean-square of the converged per-replica SEMs across four independent replicas. All propagated ΔG_{hydr} uncertainties are below 0.18 kcal mol^{-1}, confirming adequate convergence of the 5 ns production simulations.

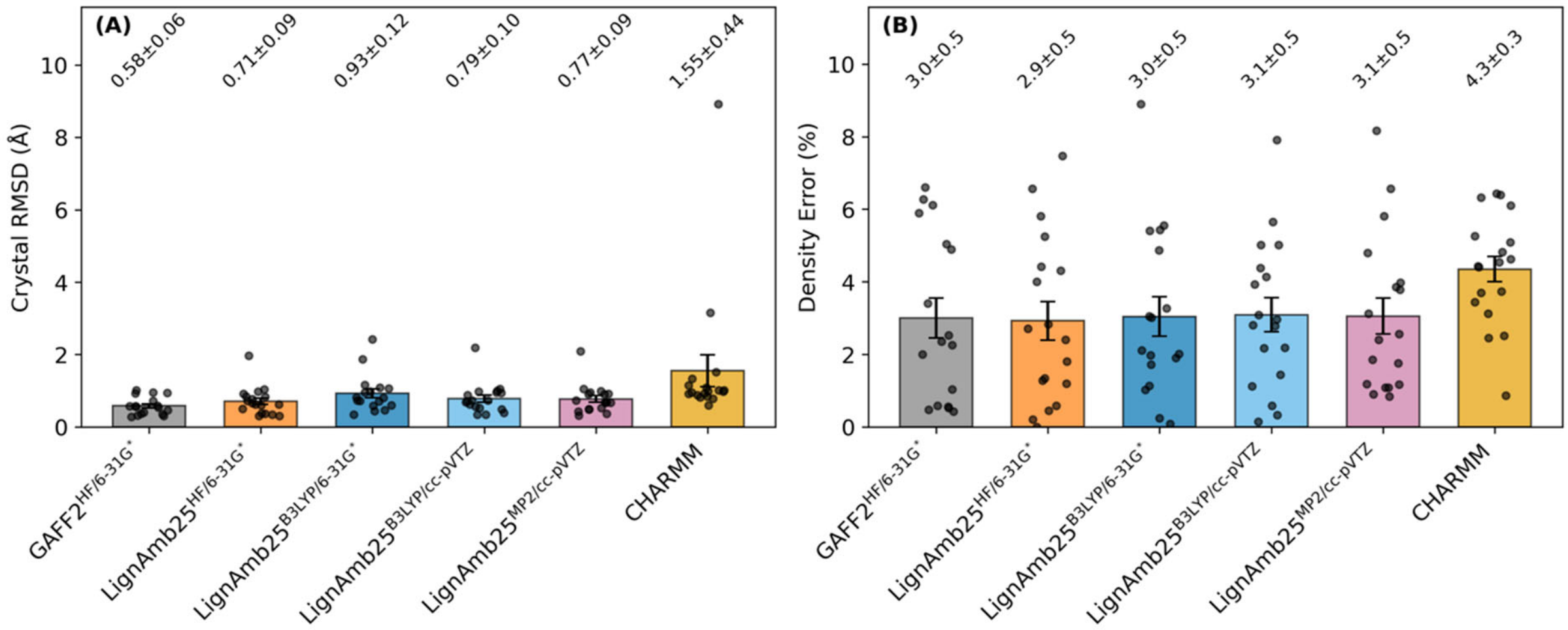

Figure S8: Crystal structure validation across force fields with a simulation protocol faithful to CHARMM-style simulations in AMBER. A) Crystal heavy-atom RMSD and (B) mean absolute density error for 18 lignin-related crystal structures. Data for GAFF2$^{HF/6\text{-}31G*}$ and all LignAmb25 force field variants were obtained by taking the last 200 frames from the last 10 ns of a 20 ns-long MD simulation over 4 independent replicas. Bar heights represent mean values; error bars indicate standard error of the mean across structures. Density error calculated as $|\rho_{calc}/\rho_{exp} - 1| \times 100\%$. Results obtained for crystal RMSD and density error are nearly identical compared to the AMBER-style simulation protocol described.

Table S1: Overview of atom types included in the LignAmb25 force field

Atom type	Target atoms
Ca	Carbonyl carbon
Cb	Beta carbon in propyl side chain
Cd	Alpha carbon bonded to aromatic ring
CD	Alpha carbon bonded to aromatic ring (alternate)
Ce	Alpha carbon with ether linkage
Cf	Gamma carbon in β-O-4 linkage (from torsion fit)
Ci	Alpha carbon in β-5 linkage (from torsion fit)
Cm	Methoxy carbon
Co	Alpha carbon with hydroxyl group
Cr	Gamma carbon in side chain
Ct	Gamma carbon in side chain (alternate)
Cx	Carbonyl carbon (alternate)
cE	Aromatic carbon with ether substituent
cH	Aromatic carbon with H (unsubstituted)
cM	Aromatic carbon with methoxy group
cm	Aromatic carbon with methoxy group (alternate)
cN	Aromatic carbon at C1
cO	Aromatic carbon with hydroxyl group
HR	Aromatic ring hydrogen
Hd	Hydrogen on alpha carbon
Hm	Methoxy hydrogen
Hq	Hydroxyl hydrogen
Hr	Hydrogen on beta/gamma carbon
Hx	Hydrogen on carbon adjacent to carbonyl
OE	Ether oxygen in aryl ether linkage
Ob	Bridging ether oxygen (β-O-4 linkage)
Oc	Phenolic/aliphatic hydroxyl oxygen
Oe	Ether oxygen (aliphatic C-O-C)
Om	Methoxy oxygen
Or	Ring ether oxygen (in furan/dioxane)
Oz	Carbonyl oxygen

Table S2: Details on CENSO settings used for identification of representative conformers from CREST-generated ensembles. All CENSO parts were run using ORCA 6.0 as the QM backend.

Stage	Functional/Basis	Solvation model	Energy Threshold
Prescreening (Part 0)	pbe-d4/def2-SV(P)	–	5.0 $kcal{\cdot}mol^{-1}$
Screening (Part 1)	r2scan-3c/def2-TZVP	SMD	3.5 $kcal{\cdot}mol^{-1}$
Optimization (Part 2)	r2scan-3c/def2-TZVP	SMD	3.0 $kcal{\cdot}mol^{-1}$
Refinement (Part 3)	wb97x-d4/def2-TZVPP	SMD	95%*

*Threshold for additive Boltzmann population beyond which conformers will be neglected

Table S3: Mean RRMSE values obtained for RESP fits at different levels of theory and charge restraint weights. The RRMSE was calculated over all force field fragments (n = 134).

Level of theory	Weight	Mean RRMSE	Standard deviation
B3LYP/6-31G*	0.0003	0.08527	0.13230
	0.0005	0.08530	0.13228
	0.0007	0.08525	0.13234
	0.001	0.08520	0.13247
B3LYP/cc-pVTZ	0.0003	0.09213	0.14215
	0.0005	0.09220	0.14214
	0.0007	0.09224	0.14218
	0.001	0.09221	0.14228
HF/6-31G*	0.0003	0.08104	0.12664
	0.0005	0.08086	0.12675
	0.0007	0.08101	0.12666
	0.001	0.08094	0.12682
MP2/cc-pVTZ	0.0003	0.09522	0.14683
	0.0005	0.09450	0.14659
	0.0007	0.09528	0.14683
	0.001	0.09531	0.14682
wB97X-D/cc-pVTZ	0.0003	0.09143	0.14159
	0.0005	0.09146	0.14158
	0.0007	0.09145	0.14160
	0.001	0.09142	0.14168

Table S4: Details on ORCA settings used for potential energy surface scans of bond stretching and bond angle bending

**SCF Convergence (tightscf)**	TolE	1e-8
	TolMAXP	1e-7
	TolRMSP	5e-9
	TolErr	5e-7
	Thresh	2.5e-11
	TCut	2.5e-12
	DFTGrid.BFCut	1e-11
	TolG	1e-5
	TolX	1e-5
	Z_Tol	1e-4
	Maxiter	1000
**Optimization convergence (tightopt)**	TolE	1e-6
	TolRMSG	3e-5
	TolMaxG	1e-4
	TolRMSD	6e-4
	TolMaxD	1e-3
	Maxiter	1000

Table S5: Details on ORCA settings used for potential energy surface scans of torsion angles. The optimization convergence criteria were less strict to reduce computational costs.

**SCF Convergence (tightscf)**	TolE	1e-8
	TolMAXP	1e-7
	TolRMSP	5e-9
	TolErr	5e-7
	Thresh	2.5e-11
	TCut	2.5e-12
	DFTGrid.BFCut	1e-11
	TolG	1e-5
	TolX	1e-5
	Z_Tol	1e-4
	Maxiter	1000
**Optimization convergence (normalopt)**	TolE	5e-6
	TolRMSG	1e-4
	TolMaxG	3e-4
	TolRMSD	2e-3
	TolMaxD	4e-3
	Maxiter	1000

Table S6: Calculated enthalpies of vaporization (ΔH_{vap}) for all compounds and parameter sets. Exp.: experimental reference value; Calc.: mean over four independent replicas; Error: Calc. – Exp; σ_{rep}: standard deviation across replicas; SEM_{block}: converged standard error of the mean from Flyvbjerg & Petersen block averaging, propagated as $\mathbf{SEM}(\boldsymbol{\Delta H_{vap}}) = \sqrt{(\mathbf{SEM}(\boldsymbol{U_g})^2 + \mathbf{SEM}(\boldsymbol{U_l})^2)}$. All values are given in kcal mol^{-1}.

Method	Compound	Exp.	Calc.	Error	σ_{rep}	SEM_{block}
GAFF2$^{AM1\text{-}BCC}$	Phenol	14.13	14.08	-0.05	0.06	0.071
	Catechol	16.73	16.60	-0.13	0.05	0.080
	Guaiacol	14.96	15.26	+0.30	0.09	0.088
	Benzene	8.10	7.49	-0.61	0.08	0.071
	Ethanol	10.11	9.57	-0.54	0.04	0.057
	Glycerol	21.63	19.97	-1.66	0.09	0.080
	Anisole	11.09	11.89	+0.80	0.11	0.083
	m-cresol	15.53	14.84	-0.69	0.07	0.084
LignAmb25$^{B3LYP/6\text{-}31G^*}$	Phenol	14.13	11.36	-2.77	0.07	0.077
	Catechol	16.73	13.81	-2.92	0.02	0.077
	Guaiacol	14.96	13.87	-1.09	0.06	0.089
	Benzene	8.10	7.09	-1.01	0.05	0.068
	Ethanol	10.11	8.90	-1.21	0.04	0.054
	Glycerol	21.63	17.21	-4.42	0.18	0.081
	Anisole	11.09	10.74	-0.35	0.06	0.084
	m-cresol	15.53	14.31	-1.22	0.07	0.083
LignAmb25$^{B3LYP/6\text{-}31G^*}$ (×1.10)	Phenol	14.13	12.64	-1.49	0.06	0.075
	Catechol	16.73	15.29	-1.44	0.01	0.080
	Guaiacol	14.96	14.99	+0.03	0.07	0.089
	Benzene	8.10	7.29	-0.81	0.04	0.072
	Ethanol	10.11	10.86	+0.75	0.11	0.054

	Glycerol	21.63	19.16	-2.47	0.06	0.085
	Anisole	11.09	11.16	+0.07	0.09	0.083
	m-cresol	15.53	16.37	+0.84	0.04	0.091
LignAmb25$^{B3LYP/6\text{-}31G*}$ (×1.20)	Phenol	14.13	14.11	-0.02	0.06	0.073
	Catechol	16.73	17.02	+0.29	0.04	0.082
	Guaiacol	14.96	16.16	+1.20	0.14	0.089
	Benzene	8.10	7.51	-0.59	0.02	0.072
	Ethanol	10.11	13.01	+2.90	0.05	0.056
	Glycerol	21.63	21.49	-0.14	0.49	0.087
	Anisole	11.09	11.76	+0.67	0.11	0.085
	m-cresol	15.53	18.45	+2.92	0.10	0.090
LignAmb25$^{B3LYP/cc\text{-}pVTZ}$	Phenol	14.13	11.04	-3.09	0.06	0.074
	Catechol	16.73	13.41	-3.32	0.09	0.077
	Guaiacol	14.96	13.68	-1.28	0.05	0.086
	Benzene	8.10	7.04	-1.06	0.05	0.067
	Ethanol	10.11	8.20	-1.91	0.06	0.051
	Glycerol	21.63	14.92	-6.71	0.12	0.078
	Anisole	11.09	10.64	-0.45	0.10	0.090
	m-cresol	15.53	13.99	-1.54	0.06	0.086
LignAmb25$^{B3LYP/cc\text{-}pVTZ}$ (×1.10)	Phenol	14.13	12.09	-2.04	0.06	0.070
	Catechol	16.73	15.08	-1.65	0.02	0.082
	Guaiacol	14.96	18.59	+3.63	0.04	0.093
	Benzene	8.10	7.30	-0.80	0.03	0.067
	Ethanol	10.11	9.89	-0.22	0.02	0.053
	Glycerol	21.63	20.94	-0.69	0.05	0.078
	Anisole	11.09	11.07	-0.02	0.04	0.084
	m-cresol	15.53	16.08	+0.55	0.03	0.081
LignAmb25$^{B3LYP/cc\text{-}pVTZ}$ (×1.20)	Phenol	14.13	13.62	-0.51	0.10	0.070
	Catechol	16.73	16.25	-0.48	0.10	0.079

	Guaiacol	14.96	17.88	+2.92	0.20	0.088
	Benzene	8.10	7.53	-0.57	0.05	0.067
	Ethanol	10.11	11.86	+1.75	0.02	0.055
	Glycerol	21.63	23.40	+1.77	0.04	0.084
	Anisole	11.09	11.59	+0.50	0.10	0.090
	m-cresol	15.53	17.75	+2.22	0.11	0.087
LignAmb25$^{HF/6\text{-}31G*}$	Phenol	14.13	12.82	-1.31	0.05	0.074
	Catechol	16.73	16.47	-0.26	0.08	0.084
	Guaiacol	14.96	14.93	-0.03	0.14	0.089
	Benzene	8.10	7.53	-0.57	0.03	0.071
	Ethanol	10.11	10.37	+0.26	0.24	0.123
	Glycerol	21.63	18.71	-2.92	0.10	0.089
	Anisole	11.09	11.45	+0.36	0.05	0.084
	m-cresol	15.53	16.84	+1.31	0.06	0.089
LignAmb25$^{MP2/cc\text{-}pVTZ}$	Phenol	14.13	11.24	-2.89	0.06	0.072
	Catechol	16.73	13.61	-3.12	0.12	0.078
	Guaiacol	14.96	13.84	-1.12	0.07	0.086
	Benzene	8.10	7.15	-0.95	0.05	0.070
	Ethanol	10.11	8.24	-1.87	0.06	0.056
	Glycerol	21.63	16.79	-4.84	0.07	0.077
	Anisole	11.09	10.82	-0.27	0.07	0.084
	m-cresol	15.53	14.57	-0.96	0.09	0.080
LignAmb25$^{MP2/cc\text{-}pVTZ}$ (×1.10)	Phenol	14.13	12.42	-1.71	0.11	0.073
	Catechol	16.73	15.04	-1.69	0.04	0.078
	Guaiacol	14.96	14.84	-0.12	0.10	0.088
	Benzene	8.10	7.46	-0.64	0.08	0.070
	Ethanol	10.11	10.00	-0.11	0.03	0.056
	Glycerol	21.63	19.14	-2.49	0.10	0.081
	Anisole	11.09	11.29	+0.20	0.08	0.084

	m-cresol	15.53	16.48	+0.95	0.05	0.081
LignAmb25$^{\omega B97X\text{-}D/cc\text{-}pVTZ}$	Phenol	14.13	11.35	-2.78	0.12	0.075
	Catechol	16.73	13.69	-3.04	0.06	0.077
	Guaiacol	14.96	17.25	+2.29	0.11	0.088
	Benzene	8.10	7.28	-0.82	0.04	0.072
	Ethanol	10.11	8.31	-1.80	0.03	0.058
	Glycerol	21.63	17.38	-4.25	0.11	0.078
	Anisole	11.09	10.87	-0.22	0.08	0.083
	m-cresol	15.53	14.34	-1.19	0.04	0.083
GAFF2$^{HF/6\text{-}31G^*}$	Phenol	14.13	12.91	-1.22	0.08	0.073
	Catechol	16.73	15.31	-1.42	0.12	0.077
	Guaiacol	14.96	15.33	+0.37	0.19	0.089
	Benzene	8.10	7.54	-0.56	0.08	0.069
	Ethanol	10.11	10.60	+0.49	0.04	0.054
	Glycerol	21.63	18.42	-3.21	0.09	0.086
	Anisole	11.09	11.46	+0.37	0.03	0.080
	m-cresol	15.53	16.65	+1.12	0.11	0.083
LF-CHARMM	Phenol	14.13	12.65	-1.48	0.05	0.071
	Catechol	16.73	16.21	-0.52	0.12	0.088
	Guaiacol	14.96	16.08	+1.12	0.06	0.12
	Benzene	8.10	7.15	-0.95	0.04	0.067
	Ethanol	10.11	10.42	+0.31	0.04	0.055
	Glycerol	21.63	16.21	-5.42	0.07	0.083
	Anisole	11.09	10.13	-0.96	0.08	0.081
	m-cresol	15.53	14.39	-1.14	0.10	0.083

Table S7: Calculated absolute hydration free energies (ΔG_{hydr}) for all compounds and parameter sets. Exp.: experimental reference value (FreeSolv database); Calc.: mean over four independent replicas; Error: Calc. – Exp.; σ_{rep}: standard error of the mean across replicas; SEM_{block}: converged standard error of the mean from Flyvbjerg & Petersen block averaging, propagated through trapezoidal integration as $\boldsymbol{SEM(\Delta G_{hydr}) = \sqrt{\sum w_i^2 \cdot SEM_i^2}}$. All values are given in kcal mol^{-1}.

Method	Compound	Exp.	Calc.	Error	σ_{rep}	SEM_{block}
GAFF2$^{AM1\text{-}BCC}$	Phenol	-6.62	-8.91	-2.29	0.12	0.12
	Guaiacol	-6.12	-7.22	-1.10	0.16	0.13
	Benzene	-0.87	-0.79	+0.08	0.18	0.11
	Ethanol	-5.01	-5.42	-0.41	0.06	0.094
	Glycerol	-9.22	-16.80	-7.58	0.09	0.123
	Anisole	-2.45	-3.45	-1.00	0.11	0.13
	m-cresol	-5.49	-8.54	-3.05	0.07	0.13
	Syringol	-7.60	-11.05	-3.45	0.08	0.14
	4-Propylguaiacol	-5.26	-5.79	-0.53	0.09	0.15
	4-hydroxybenzaldehyde	-8.83	-15.95	-7.12	0.04	0.13
	4-Propylphenol	-5.21	-7.67	-2.46	0.08	0.14
LignAmb25$^{B3LYP/6\text{-}31G*}$	Phenol	-6.62	-7.05	-0.43	0.06	0.12
	Guaiacol	-6.12	-6.83	-0.71	0.08	0.13
	Benzene	-0.87	0.40	+1.27	0.06	0.10
	Ethanol	-5.01	-4.73	+0.28	0.04	0.091
	Glycerol	-9.22	-12.05	-2.83	0.12	0.134
	Anisole	-2.45	-1.13	+1.32	0.16	0.12
	m-cresol	-5.49	-7.60	-2.11	0.15	0.13
	Syringol	-7.60	-11.42	-3.82	0.13	0.14
	4-Propylguaiacol	-5.26	-5.43	-0.17	0.11	0.15
	4-hydroxybenzaldehyde	-8.83	-12.57	-3.74	0.09	0.13

	4-Propylphenol	-5.21	-5.79	-0.58	0.06	0.15
LignAmb25$^{B3LYP/6-31G*}$ (×1.10)	Phenol	-6.62	-9.37	-2.75	0.08	0.12
	Guaiacol	-6.12	-9.29	-3.17	0.12	0.13
	Benzene	-0.87	-0.46	+0.41	0.10	0.11
	Ethanol	-5.01	-6.69	-1.68	0.07	0.097
	Glycerol	-9.22	-15.00	-5.78	0.20	0.14
	Anisole	-2.45	-2.74	-0.29	0.07	0.12
	m-cresol	-5.49	-10.40	-4.91	0.10	0.13
	Syringol	-7.60	-14.64	-7.04	0.09	0.14
	4-Propylguaiacol	-5.26	-7.93	-2.67	0.12	0.15
	4-hydroxybenzaldehyde	-8.83	-16.19	-7.36	0.10	0.13
	4-Propylphenol	-5.21	-7.64	-2.43	0.10	0.15
LignAmb25$^{B3LYP/6-31G*}$ (×1.20)	Phenol	-6.62	-12.68	-6.06	0.12	0.12
	Guaiacol	-6.12	-12.08	-5.96	0.06	0.13
	Benzene	-0.87	-1.09	-0.22	0.04	0.11
	Ethanol	-5.01	-9.06	-4.05	0.04	0.099
	Glycerol	-9.22	-18.15	-8.93	0.10	0.15
	Anisole	-2.45	-3.83	-1.38	0.06	0.12
	m-cresol	-5.49	-13.54	-8.05	0.13	0.13
	Syringol	-7.60	-18.33	-10.73	0.06	0.15
	4-Propylguaiacol	-5.26	-10.40	-5.14	0.07	0.15
	4-hydroxybenzaldehyde	-8.83	-20.58	-11.75	0.04	0.14
	4-Propylphenol	-5.21	-10.16	-4.95	0.08	0.15
LignAmb25$^{B3LYP/cc-pVTZ}$	Phenol	-6.62	-7.06	-0.44	0.06	0.12
	Guaiacol	-6.12	-7.06	-0.94	0.09	0.13
	Benzene	-0.87	0.46	+1.33	0.10	0.11
	Ethanol	-5.01	-3.98	+1.03	0.06	0.091
	Glycerol	-9.22	-10.37	-1.15	0.09	0.12
	Anisole	-2.45	-1.13	+1.32	0.08	0.12

	m-cresol	-5.49	-7.30	-1.81	0.15	0.12
	Syringol	-7.60	-11.24	-3.64	0.05	0.14
	4-Propylguaiacol	-5.26	-5.57	-0.31	0.10	0.15
	4-hydroxybenzaldehyde	-8.83	-12.49	-3.66	0.04	0.13
	4-Propylphenol	-5.21	-5.49	-0.28	0.07	0.15
LignAmb25$^{B3LYP/cc\text{-}pVTZ}$ (×1.10)	Phenol	-6.62	-9.39	-2.77	0.18	0.12
	Guaiacol	-6.12	-17.87	-11.75	0.15	0.14
	Benzene	-0.87	-0.23	+0.64	0.09	0.11
	Ethanol	-5.01	-5.61	-0.60	0.05	0.093
	Glycerol	-9.22	-16.54	-7.32	0.08	0.12
	Anisole	-2.45	-2.47	-0.02	0.07	0.12
	m-cresol	-5.49	-10.33	-4.84	0.11	0.13
	Syringol	-7.60	-14.49	-6.89	0.06	0.15
	4-Propylguaiacol	-5.26	-15.79	-10.53	0.19	0.15
	4-hydroxybenzaldehyde	-8.83	-16.14	-7.31	0.08	0.14
	4-Propylphenol	-5.21	-15.49	-10.28	0.15	0.15
LignAmb25$^{B3LYP/cc\text{-}pVTZ}$ (×1.20)	Phenol	-6.62	-12.34	-5.72	0.10	0.12
	Guaiacol	-6.12	-21.96	-15.84	0.07	0.14
	Benzene	-0.87	-1.13	-0.26	0.10	0.11
	Ethanol	-5.01	-7.98	-2.97	0.04	0.096
	Glycerol	-9.22	-20.14	-10.92	0.06	0.13
	Anisole	-2.45	-3.46	-1.01	0.10	0.13
	m-cresol	-5.49	-13.42	-7.93	0.07	0.13
	Syringol	-7.60	-18.16	-10.56	0.11	0.15
	4-Propylguaiacol	-5.26	-19.93	-14.67	0.09	0.16
	4-hydroxybenzaldehyde	-8.83	-20.44	-11.61	0.10	0.14
	4-Propylphenol	-5.21	-19.79	-14.58	0.08	0.16
LignAmb25$^{HF/6\text{-}31G^*}$	Phenol	-6.62	-7.03	-0.41	0.09	0.11
	Guaiacol	-6.12	-7.03	-0.91	0.03	0.13

	Benzene	-0.87	-1.17	-0.30	0.13	0.11
	Ethanol	-5.01	-6.49	-1.48	0.10	0.098
	Glycerol	-9.22	-14.31	-5.09	0.83	0.13
	Anisole	-2.45	-3.41	-0.96	0.01	0.12
	m-cresol	-5.49	-11.23	-5.74	0.06	0.13
	Syringol	-7.60	-11.50	-3.90	0.04	0.14
	4-Propylguaiacol	-5.26	-5.45	-0.19	0.06	0.15
	4-hydroxybenzaldehyde	-8.83	-12.35	-3.52	0.04	0.13
	4-Propylphenol	-5.21	-5.40	-0.19	0.11	0.15
LignAmb25$^{MP2/cc-pVTZ}$	Phenol	-6.62	-7.05	-0.43	0.06	0.12
	Guaiacol	-6.12	-7.27	-1.15	0.04	0.13
	Benzene	-0.87	0.10	+0.97	0.07	0.11
	Ethanol	-5.01	-4.01	+1.00	0.05	0.092
	Glycerol	-9.22	-11.91	-2.69	0.08	0.12
	Anisole	-2.45	-1.41	+1.04	0.21	0.12
	m-cresol	-5.49	-8.12	-2.63	0.12	0.12
	Syringol	-7.60	-11.09	-3.49	0.07	0.14
	4-Propylguaiacol	-5.26	-5.82	-0.56	0.09	0.15
	4-hydroxybenzaldehyde	-8.83	-12.59	-3.76	0.14	0.13
	4-Propylphenol	-5.21	-5.53	-0.32	0.06	0.16
LignAmb25$^{MP2/cc-pVTZ}$ (×1.10)	Phenol	-6.62	-7.15	-0.53	0.67	0.13
	Guaiacol	-6.12	-7.65	-1.53	0.11	0.15
	Benzene	-0.87	0.47	+1.34	0.13	0.12
	Ethanol	-5.01	-5.42	-0.41	0.05	0.10
	Glycerol	-9.22	-14.22	-5.00	0.08	0.13
	Anisole	-2.45	-0.86	+1.59	0.02	0.13
	m-cresol	-5.49	-9.11	-3.62	0.13	0.14
	Syringol	-7.60	-12.71	-5.11	0.16	0.16
	4-Propylguaiacol	-5.26	-5.22	+0.04	0.08	0.17

	4-hydroxybenzaldehyde	-8.83	-14.41	-5.58	0.08	0.14
	4-Propylphenol	-5.21	-4.55	+0.66	0.87	0.17
LignAmb25$^{\omega B97X\text{-}D/cc\text{-}pVTZ}$	Phenol	-6.62	-6.92	-0.30	0.09	0.12
	Guaiacol	-6.12	-12.75	-6.63	0.12	0.14
	Benzene	-0.87	-0.12	+0.75	0.05	0.10
	Ethanol	-5.01	-4.19	+0.82	0.05	0.093
	Glycerol	-9.22	-11.96	-2.74	0.43	0.12
	Anisole	-2.45	-1.89	+0.56	0.11	0.12
	m-cresol	-5.49	-7.97	-2.48	0.15	0.12
	Syringol	-7.60	-11.34	-3.74	0.05	0.14
	4-Propylguaiacol	-5.26	-11.34	-6.08	0.09	0.15
	4-hydroxybenzaldehyde	-8.83	-12.25	-3.42	0.09	0.13
	4-Propylphenol	-5.21	-11.20	-5.99	0.05	0.15
GAFF2$^{HF/6\text{-}31G*}$	Phenol	-6.62	-7.06	-0.44	0.04	0.12
	Guaiacol	-6.12	-7.35	-1.23	0.04	0.13
	Benzene	-0.87	-1.05	-0.18	0.13	0.11
	Ethanol	-5.01	-6.48	-1.47	0.07	0.096
	Glycerol	-9.22	-14.93	-5.71	0.12	0.12
	Anisole	-2.45	-3.41	-0.96	0.06	0.12
	m-cresol	-5.49	-10.95	-5.46	0.11	0.13
	Syringol	-7.60	-9.99	-2.39	0.08	0.14
	4-Propylguaiacol	-5.26	-5.37	-0.11	0.07	0.15
	4-hydroxybenzaldehyde	-8.83	-13.29	-4.46	0.07	0.13
	4-Propylphenol	-5.21	-5.33	-0.12	0.15	0.15
LF-CHARMM	Phenol	-6.62	-6.99	-0.37	0.08	0.12
	Guaiacol	-6.12	-8.54	-2.42	0.81	0.14
	Benzene	-0.87	-0.53	+0.34	0.10	0.11
	Ethanol	-5.01	-8.31	-3.30	0.07	0.10
	Glycerol	-9.22	-14.60	-5.38	0.15	0.13

Anisole	-2.45	-3.06	-0.61	0.04	0.13
m-cresol	-5.49	-7.66	-2.17	0.15	0.13
4-Propylguaiacol	-	-	-	-	-
4-hydroxybenzaldehyde	-	-	-	-	-
4-Propylphenol	-	-	-	-	-

Table S8: Comparison of mean crystal properties after simulation using the established lignin forcefield for CHARMM (CHARMM), the GAFF2 force field parameters with LignAmb25 RESP charges (GAFF2), and the MP2/cc-pVTZ-based LignAmb25 force field (LignAmb25).

Name (CSD Code)	ρ ratio $\left(\frac{\rho_{sim}}{\rho_{exp}}\right)$[a]			RMSD^C [Å][b]			RMSD^M [Å][c]		
	LignAmb[d]	CHARMM	GAFF2	LignAmb	CHARMM	GAFF2	LignAmb	CHARMM	GAFF2
Catechol (CATCOL13)	1.0657(2)	0.9687(2)	1.0603(3)	0.370(7)	0.977(8)	0.411(8)	0.04(0)	0.05(1)	0.04(0)
p-Hydroxy benzaldehyde (PHBALD11)	1.0240(8)	0.9755(6)	1.0504(8)	1.056(1)	1.09(2)	0.937(1)	0.10(0)	0.11(3)	0.10(0)
Vanilin (YUHTEA01)	1.0398(3)	0.9749(2)	1.0662(4)	0.732(8)	1.331(8)	0.542(1)	0.09(0)	0.10(2)	0.09(0)
Vanilin (YUHTEA03)	1.0312(7)	1.061(1)	1.0591(7)	0.911(2)	1.52(3)	0.716(2)	0.12(0)	0.14(4)	0.13(0)
Siringaldehyde (IZALAW)	0.9183(7)	0.9491(4)	0.9225(7)	0.904(1)	8.92(1)	0.897(1)	0.14(0)	0.19(4)	0.19(0)
Coniferylaldehyde (SIPKEH)	0.9544(7)	0.9913(6)	0.9698(9)	0.632(1)	3.16(2)	0.635(2)	0.17(0)	0.5(1)	0.19(0)
Vanilic acid (CEHGUS)	0.9621(8)	0.9360(4)	0.9366(8)	0.682(1)	1.01(1)	0.808(1)	0.15(0)	0.15(4)	0.15(0)
Ferulic acid (GASVOL01)	0.9824(3)	0.9557(2)	1.0042(3)	0.322(4)	0.837(6)	0.276(3)	0.14(0)	0.11(3)	0.14(0)
G-βO4-G (RABWUM)	0.9950(3)	0.9630(2)	0.9948(4)	1.367(2)	0.592(7)	0.310(5)	0.37(1)	0.20(4)	0.15(0)
G-βO4-G (SIPPEM)	0.9613(6)	0.9538(3)	0.9704(5)	0.943(4)	0.93(1)	0.526(9)	0.33(1)	0.36(9)	0.23(0)
S-βO4-G (VADDOT)	1.0386(7)	0.9546(3)	1.0498(6)	0.671(1)	1.15(1)	0.579(1)	0.30(0)	0.29(7)	0.26(0)
S-βO4-S (SAZHEG)	0.9899(1)	0.9474(4)	1.0062(9)	0.988(1)	0.95(1)	0.561(2)	0.53(0)	0.25(6)	0.28(1)
S-βO4-S (FOCGUA)	1.0581(2)	0.9559(1)	1.0628(3)	0.488(7)	0.917(7)	0.463(7)	0.17(0)	0.19(4)	0.21(0)
S-βO4-S (IDIKIP)	1.0138(6)	0.9480(2)	1.0301(4)	1.185(8)	0.84(1)	0.568(9)	0.39(1)	0.19(4)	0.25(0)
G-ββ-G (INELIW)	0.9814(4)	0.9626(2)	0.9591(1)	0.433(7)	0.786(9)	1.686(5)	0.14(0)	0.11(7)	0.53(1)
G-ββ-G (INELIW01)	0.9882(4)	0.9656(2)	0.9771(4)	0.904(4)	0.82(2)	0.971(6)	0.11(0)	0.12(9)	0.44(0)
G-ββ-G (FAFXUF)	0.9744(7)	0.9368(4)	0.8976(2)	0.440(9)	0.99(1)	3.365(8)	0.21(0)	0.19(4)	1.45(2)
G-β5-G (FUMVUE)	1.0117(7)	0.9357(3)	1.0226(6)	0.684(1)	1.01(1)	0.553(1)	0.23(0)	0.25(7)	0.20(0)
DBDO (TUGWAT)	1.0111(5)	0.9518(4)	1.0048(4)	0.528(5)	0.88(2)	0.362(6)	0.39(0)	0.29(4)	0.22(0)

[a]Density ratio calculated from the simulated (ρ_{sim}) and experimentally measured crystal structure (ρ_{exp})

[b]RMSD^C calculated as the RMSD of the simulated relative to the deposited crystal structure

[c]RMSD^M calculated as the average intramolecular RMSD of the molecule in the simulated relative to the deposited crystal structure

[d]Data for GAFF2 and LignAmb25 were obtained from 20 independent replica simulations by taking the last 200 frames from the simulation trajectory, corresponding to the last 10 ns of the total 20 ns simulated, whereas data for CHARMM were taken as reported from the source publication. Uncertainty in the last digit is reported in parentheses.
